# On the survival of the quantum depletion of a condensate after release from a magnetic trap

**DOI:** 10.1038/s41598-022-16477-9

**Published:** 2022-08-01

**Authors:** J. A. Ross, P. Deuar, D. K. Shin, K. F. Thomas, B. M. Henson, S. S. Hodgman, A. G. Truscott

**Affiliations:** 1 Research School of Physics, Australian National University, Canberra, 0200 Australia; 2 Institute of Physics, Polish Academy of Sciences, Aleja Lotników 32/46, 02-688 Warsaw, Poland

**Keywords:** Condensed-matter physics, Quantum physics, Physics

## Abstract

We present observations of the high momentum tail in expanding Bose–Einstein condensates of metastable Helium atoms released from a harmonic trap. The far-field density profile exhibits features that support identification of the tails of the momentum distribution as originating in the in-situ quantum depletion prior to release. Thus, we corroborate recent observations of slowly-decaying tails in the far-field beyond the thermal component. This observation is in conflict with the hydrodynamic theory, which predicts that the in-situ depletion does not survive when atoms are released from a trap. Indeed, the depleted tails even appear stronger in the far-field than expected before release, and we discuss the challenges of interpreting this in terms of the Tan contact in the trapped gas. In complement to these observations, full quantum simulations of the experiment show that, under the right conditions, the depletion can persist into the far field after expansion. Moreover, the simulations provide mechanisms for survival and for the the large-momentum tails to appear stronger after expansion due to an acceleration of the depleted atoms by the mean-field potential. However, while in qualitative agreement, the final depletion observed in the experiment is much larger than in the simulation.

## Introduction

In the Bogoliubov description of an ultracold interacting superfluid, the ground state is composed of a macroscopically-occupied condensate and correlated particle pairs due to s-wave interactions between constituent particles^1,2^. A consequence of these pairs is that excited single-particle modes are populated even at zero temperature. This is the *quantum depletion* of the condensate and presents as an occupation of single particle modes, which at large momentum *p* decays^3,4^ like $p^{-4}$.

Since the realization of atomic Bose–Einstein condensates (BECs) there has been considerable experimental^2,5–19^ and theoretical^4,20–33^ interest in the Bogoliubov theory^2,17,32–34^ (and quantum depletion specifically^7,10–13,35^). In contrast to the case of liquid helium, where the depleted fraction is large (of order 93% of the fluid^36–38^) due to the strong interparticle interactions, the depletion is generally very small (less than 1%^7,12^) in weakly-interacting dilute gases. The intimately related thermodynamic (Tan’s) contact has also received growing attention^4–9,14–16,18–20,22–31,35,39–42^, in part due to Tan’s proof that the contact is directly related to the amplitude of the $p^{-4}$ tail^39^.

Experiments examining the large-momentum tails have typically employed Feshbach resonances to enhance interactions in ultracold gases and produce a depleted fraction visible in the far-field with standard imaging techniques, but the power-law tails have proven elusive^8,9^ in this regime. A handful of theories have emerged^20–22^ which elucidate the role played by many-body interactions in modifying the momentum distribution during the evolution following a quench to a large scattering length. A very recent experiment^43^ was able to detect pairs of atoms with anticorrelated momenta in the far-field by use of an optical lattice to create a BEC in a high-density, strongly-interacting regime. However, measurements in the weakly-interacting regime have returned unexpected results. A previous experiment reported the presence of power-law-like tails in the far-field distribution after releasing a BEC of metastable helium from a harmonic optical trap^7^. This was surprising because conventional wisdom argues that the density decreases adiabatically during expansion (even when the trap release is non-adiabatic), motivating a hydrodynamic approximation wherein the tails are predicted to vanish^10,23^. Moreover, the tails were reported to be approximately six times heavier than predicted by Bogoliubov theory. It is important to verify the anomaly and understand its origin because far-field measurements play a central role in the study of ultracold gases. The prospect of extracting correlated depleted pairs from a zero-temperature ground state is also conceptually, and possibly technologically, interesting in itself.

To these ends, we measure the far-field momentum distribution of a BEC of metastable helium (He$^*~$) expanding from a harmonic trap. We observe tails in the large-momentum part of the (far-field) condensate wavefunction whose amplitude depends nonlinearly on the condensate population, and whose density profile is consistent with a $p^{-4}$-like power law decay, in a manner consistent with the predictions of the Tan and Bogoliubov theory. Specifically, the amplitude of the far-field momentum tails is shown to have a linear relationship with the product of the condensate population and peak density, as predicted by both theories. However, there is a quantitative difference in amplitude between the predicted and measured values. Our measurements are complemented by numerical simulations of the dynamics of the momentum distribution after the trap release using a Stochastic Time-Adaptive Bogoliubov (STAB) method in the positive-P framework^33,44^. These demonstrate a mechanism for survival associated with the non-adiabatic release of the trap, and suggest that the depleted particles acquire additional kinetic energy from the mean-field energy of the condensate during the subsequent adiabatic expansion. These factors result in an amplification of the density of the far-field momentum tails relative to the in-situ values by a factor of up to about two, and are absent from the hydrodynamic approximation. However, even taking these effects into account, the amplitude of the measured tails is still significantly larger than expected from the simulations.

### Quantum depletion of the condensate by contact interactions

Before presenting our results, let us introduce the central theoretical assumptions and predictions relevant for this work. The Hamiltonian of a homogeneous system of interacting bosons can be written in terms of plane-wave field operators $\hat{a_{{\textbf {k}}}}$, labeled by the wavevector ${{\textbf {k}}}={{\textbf {p}}}/\hbar$, and diagonalized by the Bogoliubov transformation to a free Bose gas of collective excitations through the operator transformation ${\hat{b}}_{{{\textbf {k}}}}^\dagger = u_k {\hat{a}}_{{\textbf {k}}}^\dagger + v_k {\hat{a}}_{-{{\textbf {k}}}}$^1,45^. The collective excitations are superpositions of particles with opposite momenta^2^, and the $u_k$ and $v_k$ coefficients are given by

1 $$\begin{aligned} u_k&= \cosh \theta _k, \qquad v_k = \sinh \theta _k,\end{aligned}$$

2 $$\begin{aligned} \theta _k&= \frac{1}{2}\log \frac{\hbar ^2k^2/2m}{\varepsilon (k)} <0, \end{aligned}$$

where the denominator is the quasiparticle dispersion

3 $$\begin{aligned} \varepsilon (k) = \sqrt{\left( \frac{\hbar ^2k^2}{2m}\right) ^2 + gn\frac{ \hbar ^2k^2}{m}}, \end{aligned}$$

determined by the particle density *n*, the atomic mass *m*, and the effective interaction strength $g=4\pi \hbar ^2a/m$, where *a* is the s-wave scattering length^3,45^. In the non-interacting ($a\rightarrow 0$) limit, $u_k=1$ and $v_k=0$, so the transformation reduces to the identity and the dispersion is that of free particles. The occupation of single-particle momentum modes can be found using the inverse transformation and is given by

4 $$\begin{aligned} \rho ({{\textbf {k}}})&= \langle {\hat{a}}_{{\textbf {k}}}^\dagger {\hat{a}}_{{\textbf {k}}}\rangle, \end{aligned}$$

5 $$\begin{aligned}\rho ({{\textbf {k}}})&=\left( u_{k}^{2}+v_{k}^{2}\right) \langle {\hat{b}}_{{{\textbf {k}}}}^{\dagger }{\hat{b}}_{{{\textbf {k}}}}\rangle + v_{k}^{2}, \end{aligned}$$

wherein the quasiparticle population statistics follow the canonical ensemble as^3,7^ $\langle {\hat{b}}^\dagger _{{\textbf {k}}}{\hat{b}}_{{\textbf {k}}}\rangle = (\exp [\varepsilon (k)/k_B T]-1)^{-1}$. At finite temperatures, quasiparticle modes are thermally populated and deplete the condensate. Even at zero temperature, when the thermal fraction vanishes, the $v_k^2$ term in Eq. (5) persists giving a zero-temperature population of excited particles^4,7,46^ which decays as^3,7,45^ $\lim _{k\rightarrow \infty }\rho ({{\textbf {k}}})\propto k^{-4}$. Bogoliubov’s theory makes accurate predictions of the total depleted population in ultracold atomic Bose-Einstein condensates (BECs)^10,12^ and exciton-polariton condensates in solid substrates^11^.

In the case of a harmonically trapped gas, one can employ the local-density approximation (LDA) to compute the amplitude of the $k^{-4}$ tail by integrating $v_k^2$ across a Thomas–Fermi distribution^7^. One can also compute the expected amplitude of the tails using thermodynamic relations between the condensate mean-field energy and momentum distribution: The amplitude of the tails was shown by Tan to be exactly the quantity called the *contact*, which is proportional to the derivative of the energy with respect to the s-wave scattering length^25,39^. For a Bose gas at equilibrium in a harmonic trap, the tail amplitude can be calculated using Tan’s original theorems. The two-body *contact intensity* is defined by^25,39^

6 $$\begin{aligned} C = \lim _{k\rightarrow \infty }k^4\rho (k), \end{aligned}$$

which is related to the total contact (or just *contact*) ${\mathscr {C}} = \int C({{\textbf {r}}}) d^3 {{\textbf {r}}}$. The contact can be derived from the total energy *E* through the *adiabatic sweep theorem*^40^,

7 $$\begin{aligned} {\mathscr {C}} = \frac{8\pi m a^2}{\hbar ^2}\frac{\partial E}{\partial a}. \end{aligned}$$

Applying this to the Thomas–Fermi energy of a harmonically trapped condensate,

8 $$\begin{aligned} \frac{E}{N_0} = \frac{5}{7}\mu = \frac{5}{7} \frac{\hbar {\bar{\omega }}}{2} \left( \frac{15 N_0 a}{a_{{\text {HO}}}}\right) ^{2/5}, \end{aligned}$$

where $a_{{\text {HO}}} = \sqrt{\hbar /(m {\bar{\omega }})}$ is the harmonic oscillator length and ${\bar{\omega }}=\root 3 \of {\omega _x \omega _y \omega _z}$ is the geometric trapping frequency^3,45^, leads to the expression

9 $$\begin{aligned} {\mathscr {C}} = \frac{8\pi }{7} \left( 15^{2}(a N_0)^{7} \left( \frac{m {\bar{\omega }}}{\hbar }\right) ^{6}\right) ^{1/5}, \end{aligned}$$

for the contact and thus the asymptotic momentum (density) distribution *n*(*k*) of the in-situ condensate is,

10 $$\begin{aligned} \lim _{k\rightarrow \infty } n(k) = {\frac{{\mathscr {C}}}{k^4}} = \frac{64\pi ^2a^2}{7} \frac{N_0n_0}{k^4}, \end{aligned}$$

which depends on the peak density of the harmonically trapped condensate, in turn given by

11 $$\begin{aligned} n_0 = \frac{1}{8 \pi }\left( (15N_0)^2 \left( \frac{m {\bar{\omega }}}{\hbar \sqrt{a}}\right) ^{6}\right) ^{1/5}. \end{aligned}$$

Note that hereon we refer to the momentum distribution $n({{\textbf {k}}})$ rather than the occupation numbers $\rho ({{\textbf {k}}}) = n(k) d^3{{\textbf {k}}}/(2\pi )^3$, and that the total number of atoms in this normalisation is $N=\frac{1}{(2\pi )^3}\int d^3 {{\textbf {k}}}\, n({{\textbf {k}}})$.

## Results

### Experimental measurements

Our experimental sequence began with BECs consisting of between $2\times 10^5$ and $5 \times 10^5$ $^4$ $\hbox {He}^*$ atoms, spin-polarized in the $2^{3}S_1(m_J=1)$ state and cooled to $\sim$ 300 nK by forced evaporative cooling in a harmonic magnetic trap generated by field coils in a Bi-planar Quadrupole Ioffe configuration^47^. The trap was then switched off with a 1/*e* time of ${\tau _{\mathrm{release}}}\approx 38\,\upmu$s. The condensates were allowed to expand for 2 ms before we transferred about one quarter of the initial $m_J=1$ condensate into the magnetically insensitive $m_J=0$ state with a radio-frequency (RF) Landau–Zener sweep to preserve it against distortion by stray magnetic fields during the free fall to the detector. We deflected the $m_J=\pm 1$ clouds away from the detector with a Stern–Gerlach scheme immediately after the RF pulse by switching on a magnetic field. The centre of mass of the cloud then impacts on the detector after a $\tau = 417$ ms time of flight following the trap switch-off.

Investigations of the quantum depletion in He$^*~$are challenging because the absence of a known Feshbach resonance precludes control over the contact ${\mathscr {C}}\propto ((a N_0)^7{\bar{\omega }}^6)^{1/5}$ via the scattering length *a*. Given the small fixed $a=7.512$ nm^48^, we test the validity of Eq. (10) for describing the far-field by varying the density of the gas, $n\propto \left( N_{0}{\bar{\omega }}^3\right) ^{2/5}$ (c.f. Eq. (10)). To achieve this we used two trap configurations with $(\omega _x,\omega _y,\omega _z)\approx 2\pi (45,425,425)$ Hz (geometric mean ${\bar{\omega }} = 2\pi \cdot 201$ Hz) and $\approx \,2\pi (71,902,895)$ Hz (${\bar{\omega }} = 2\pi \cdot 393$ Hz) where the frequencies are known within 1% the (weak) axis of symmetry is horizontal. We varied the endpoint of the evaporative cooling ramp to adjust the number of atoms in the condensate.

Our experiment uses single-particle detection with multichannel-plate and delay-line detector (MCP-DLD) stacks^49^ after a long time of flight (hence in the far-field regime) enabled by the large (19.8 eV) internal energy^50^ of the metastable $2^{3}S_1$ state, $\hbox {He}^*$. The unique capabilities of such setups have permitted the observation of many-body momentum correlations^51,52^ and the Hanbury Brown-Twiss effect in both condensed^49,53–57^ and quantum depleted atoms^13,35^. We are thus able to reconstruct the full single-atom momentum distribution in three dimensions and examine the dilute far-field momentum tails of the $m_J=0$ clouds in detail.

**Figure 1:**
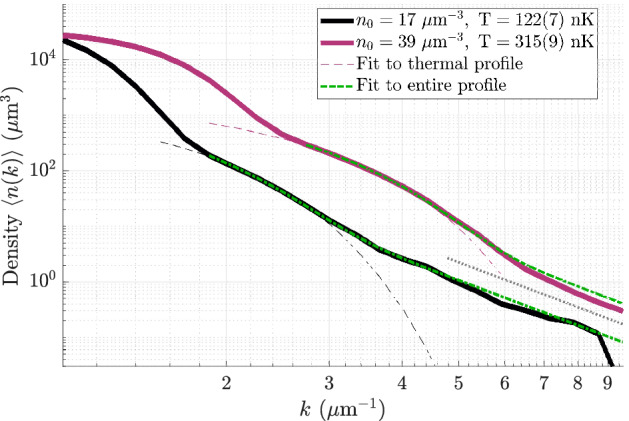
The measured far-field density of particle momenta from two trap configurations (black and magenta). Three regions are shown: At low *k* the parabolic distribution of the BEC dominates. For larger *k*, the thermal parts (fits shown by dashed lines) decay super-exponentially as $e^{-k^2}$. For even larger *k*, these give way to the surmised quantum depletion region. A combined fit of the form $n_T(k) + C_4/k^4$ (green dot-dash lines) yields temperatures consistent with the thermal fit and also an amplitude $C_4$ of the depleted tail. The grey dotted line is a guide to the eye showing a $k^{-4}$ decay. Due to constraints of the detector geometry (see Supplementary Materials for details), these profiles were integrated over two spherical segments, each subtending an angle of $\pi /6$ radians with the $\pm z$ axes (see also Fig. 5). The detector shows signs of saturation for low $k (\lesssim 1.5~\mu {{\text {m}}}^{-1}$). These factors imply the total area under the curves is less than the average number of trapped atoms.

In Fig. 1 we show the empirical far-field density *n*(*k*) for two data collection runs at the extreme values of $n_0$ we used. The black (purple) correspond to condensates with an average of $3.5 \times 10^5 (4.5 \times 10^5)$ atoms and a thermal fraction of 9% (10%). The (geometric) trap frequencies were $2\pi \cdot 201$ and $2\pi \cdot 393$ Hz, and the healing length $\xi = \hbar /\sqrt{2mgn_0}$ at the center of these clouds were $56~\mu {{\text {m}}}$ and $36~\mu {{\text {m}}}$, respectively. The three regimes of the condensate, thermal depletion, and quantum depletion span over five orders of magnitude in density. The thermal part of the distribution is well fitted by the momentum distribution of an ideal Bose gas^58^

12 $$\begin{aligned} \frac{n_T(k)}{{(2\pi )^3}} =\frac{N_T}{\zeta (3)} ~\left( \frac{\lambda _{dB}}{2\pi }\right) ^3 g_{3/2}\left( \exp \left( -\frac{k^2 \lambda _{dB}^2}{4\pi }\right) \right) , \end{aligned}$$

wherein the thermal de Broglie wavelength $\lambda _{dB} = \sqrt{2\pi \hbar ^2/(m k_B T)}$ yields an estimate of the temperature *T* which ranges from 100 to 320 nK in our experiments. Here, $g_{3/2}(\cdot )$ is the standard Bose integral, $\zeta (\cdot )$ is the Riemann zeta function, and $N_T$ is the number of atoms in the thermal component. Note that for a non-interacting gas in the thermodynamic limit, the number of thermal atoms is simply ${N_T^{\mathrm{id}}} = \zeta (3)(k_B T / \hbar {\bar{\omega }})^3{=\eta _T N}$, but for our condensates the critical temperature is reduced by $\approx 20\%$ by interactions^3,45^. We account for this and the approximately twofold increase in the thermal fraction $\eta _T$ (relative to the non-interacting case) by explicitly using $N_T$ as a fit parameter. At larger values of momentum, where the thermal component makes a negligible contribution, there appears a slow decay which we identify as the quantum depletion.

### Analysis of experimental results

A standard approach to analysing the empirical momentum density would be to proceed with a routine fit of the *k*-space histogram with an additional term of the form $C_\alpha /k^\alpha$ to estimate the parameters of the purported quantum-depleted tail. If we augment the thermal fit function (Eq. (12)) with a power-law term as per

13 $$\begin{aligned} {n(k)} = n_T(k) + \frac{C_{\alpha }}{k^{\alpha }}, \end{aligned}$$

and leave $\alpha$ as a free parameter, the average exponent over all runs is 4.2(4). For comparison, the prior work^7^ reported power-law tails with an exponent 4.2(2). At first glance, one could simply determine the amplitude of the tails by fixing the exponent to 4, and if we do so, we find an average $C_{\alpha =4}$ which is approximately 8(2) times greater than the coefficient predicted by Eq. (10), and in general agreement with Ref.^7^. However, as we detail in the supplementary materials, the covariance of the fit parameters *C* and $\alpha$, coupled with the exponential relationship to the independent variable *k*, means that this gives a significant underestimate of the uncertainty in $C_\alpha$. In general, fitting power laws to data is known to be prone to return biased estimates of parameters and to drastically under-report uncertainties^59,60^, especially when data is available over less than a couple of decades of dynamic range.

Below we present a number of lines of evidence that support the identification of these tails as originating in the quantum depletion, but we also argue there is not sufficient reason to assume that the fit with a fixed $\alpha =4$ is appropriate. The main reason for the latter is that the far-field momentum distribution is known to be a modification of the in-situ distribution due to the dispersal of the condensate mean-field energy into kinetic energy. Even neglecting this effect, it is not a given that the far-field distribution could be modified in such a way as to simply increase the amplitude of the tails without otherwise altering the functional form (i.e. the exponent in this case). As the authors of Ref.^59^ note, “In practice, we can rarely, if ever, be certain that an observed quantity is drawn from a power-law distribution. The most we can say is that our observations are consistent with the hypothesis that *x* is drawn from [...] a power law”. Indeed, this analysis does show that the far-field momentum distribution is consistent with a power-law exponent $3.8\le \alpha \le 4.6$, but the data at hand cannot precisely determine the exponent $\alpha$ (nor $C_\alpha$), as detailed in the supplement.

**Figure 2:**
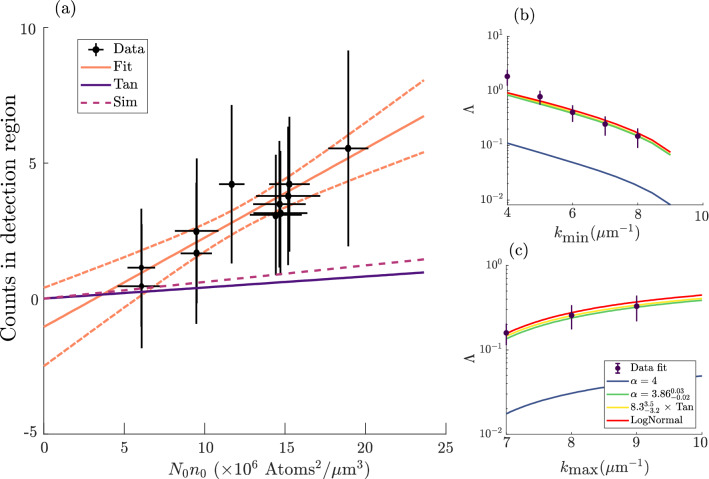
Population of momentum tails, including excess compared to Tan–Bogoliubov theory. (**a**) The product $N_0n_0$ is a linear predictor of the number of counts within the region $(k_{{\text {min}}}=6~\mu {{\text {m}}}^{-1},k_{{\text {max}}}=10~\mu {{\text {m}}}^{-1})$, consistent with Eq. (10) (solid orange line, dashed lines 95% CI). The gradient $\Lambda$ in Eq. (15) can be predicted using Eq. (10) ($\Lambda _{\mathrm{pred}}$ solid purple line) but this disagrees with the experiment by a factor of about 8. Our simulations (dashed line, CE in Fig. 3a) show an increase in counts after release but by less than in the experiment. In (**b,c**) linear fits to the experimental data yield $\Lambda _{\mathrm{fit}}$ (points) which vary with the choice of *k* bounds (fixing $k_{{\text {max}}}=10\,\upmu {{\text {m}}}^{-1}$ in (**b**) and $k_{{\text {min}}}=6\,\upmu {{\text {m}}}^{-1}$ in (**c**)). ﻿For comparison, we show predictions of $\Lambda$ based directly on Eq. (10) ($\Lambda _{\mathrm{pred}}$, blue, $n(k)={\mathscr {C}}/k^4$), along with the predictions from Eq. (15) using a density function $n(k)={\mathscr {A}\mathscr {C}/k^4}$ that has an additional prefactor ${\mathscr {A}}=8(3)$ (green) and one that has a modified exponent of $\alpha =3.86(2)$ via $n(k)={\mathscr {C}}/k^{\alpha }$ (yellow). A log-normal distribution produces nearly identical predictions (red, offset vertically for visibility). Quoted error estimates correspond to 95% CI of the fit parameters. In (**b**), the deviation from the predictions at $k_{{\text {min}}}\lesssim \,6~\mu {{\text {m}}}^{-1}$ is because the collection area starts to overlap with the thermal cloud.

Rather than explicitly enforce a power law decay assumption, we focus on another observable which can be readily measured and predicted: The number of atoms whose wavevector has a modulus in the interval $k\in (k_{{\text {min}}}, k_{{\text {max}}})$,

14 $$\begin{aligned} N_{k_{{\text {min}}},k_{{\text {max}}}} =\frac{{\mathscr {C}}}{2\pi ^2}\left( \frac{1}{k_{{\text {min}}}}-\frac{1}{k_{{\text {max}}}}\right) . \end{aligned}$$

Note that the integral of *n*(*k*) is most easily performed in spherical coordinates and requires the Jacobian $(2\pi )^{-3}{d^3}{{\textbf {k}}}$ to ensure normalization. For fixed $k_{{\text {min}}}$ and $k_{{\text {max}}}$, Eq. (14) has the form

15 $$\begin{aligned} N_{k_{{\text {min}}},k_{{\text {max}}}} = \Lambda N_0n_0, \end{aligned}$$

(c.f. Eq. (10)). We can thus test Eq. (15) directly by measuring the number of counts detected in the interval $(k_{{\text {min}}},k_{{\text {max}}})$ after producing a BEC of $N_0$ atoms with peak density $n_0$. A key advantage of this method is that theoretical assumptions (such as the exponent of the power law) are not required when analysing the experimental data, but only when calculating the (independent) prediction, i.e. the data processing is essentially theory-free.

Under the null hypothesis (based on the hydrodynamic theory) that the in situ depletion does not survive the expansion, $\Lambda =0$. Further, most types of technical noise masquerading as high energy tails would be expected to not follow the $N_0n_0$ scaling and give at best a poor correlation with Eq. (15). As we show in Fig. 2, a linear fit of the form ${\hat{N}}_{k_{{\text {min}}},k_{{\text {max}}}} = \Lambda _{{\text {fit}}} n_0 N_0 + \beta$ yields an intercept consistent with zero ($\beta$=− 0.9, 95% CI (− 3.1, 1.2)) and a good correlation ($r^2\approx 0.8$, $p=1\times 10^{-3}$), providing evidence supporting the expected linear relationship with $n_0N_0$, and against the high energy tails being due to some technical noise. The correlation coefficient between the variables $N_{k_{\mathrm{min}},k_{\mathrm{max}}}$ and $N_0n_0\propto (N_0^7{\bar{\omega }}^6)^{1/5}$ is 0.9. We conclude that the product $N_0n_0$ is a predictor of the high energy population, which is consistent with Eq. (10).

For comparison, a linear fit proves that the atom number $N_0$ itself is a poor predictor of the detected number ($r^2 = 0.05~,p = 0.54$), as is the central density $n_0$ alone ($r^2 = 0.4~,p = 0.04$). Accordingly, the particular nonlinear scaling of detected counts with the predictor $N_0n_0$ is concordant with the tails’ originating in the quantum depletion, and inconsistent with any technical noise that we know of.

The gradient $\Lambda _{{\text {fit}}}$ is of particular interest because it can be predicted using Eq. (14). Given a region of interest (ROI) over which we count atoms, one can calculate $\Lambda _{{\text {pred}}} = 32 \varepsilon a^2(k_{{{\text {min}}}}^{-1}-k_{{{\text {max}}}}^{-1})/7$, where $\varepsilon$ is the total detection efficiency. In our experiment, $\varepsilon \approx 0.23(5)\%$ (see “Methods” section for details). In comparison with the predicted value $\Lambda _\mathrm {pred} = 2.7(6) \times 10^{-7}$ (units of $\upmu {{\text {m}}}^3$/atom), we find that the empirical fit disagrees with the predicted slope by a factor of ${\mathscr {A}}_\mathrm {exp}=\Lambda _{{\text {fit}}}/\Lambda _{{\text {pred}}}= 8.3$, 95% CI (5.5, 11), which rules out the null hypothesis.

While this result may appear to restate the previously-mentioned fitting approach, which gave an increase of the $C_4$ coefficient by a factor of 8(2), it in fact complements it. In this case the overpopulation of the tails is directly measured without any recourse to assumptions about power-law behaviour in the data itself. The direct comparison of the populations in a given *k*-interval allows for an independent comparison between the prediction and measured result and seeks to simply answer the question does the data satisfy the most general model of quantum depletion proposed by Eq. (15).

In summary, there are three robust conclusions that can be drawn from the data. *First*, the population in the high-momentum tails depends linearly on the product $n_0N_0$, which is a prediction of the Tan and Bogoliubov theories and not readily associated with any other known physical process. *Second*, there are some 8(3) times as many particles in the far-field, high-momentum tails as would be expected to be found in the same interval of the in-situ distribution. *Third*, the data is consistent with power-laws with exponents in the range $3.8 \le \alpha \le 4.6$.

Interestingly, if one were to take the first observation as sufficient (and indeed independent) evidence to identify the tails with the quantum depletion *and* assume a power-law decay of the form $C_4 k^{-4}$, then one obtains a value of $C_4$ that is consistent with the regression against $n_0N_0$. While this is evidence that the $\alpha =4$ hypothesis is not inconsistent with the data, it is essentially the same as calculating *C* from the results of the linear regression by assuming $\alpha =4$. In Fig. 2b,c, as a counterpoint to power law fits over $k_{\mathrm{min, max}}$ shown in blue, green and yellow, we also show, in red, predictions obtained by assuming log-normally distributed *k* with parameters $(\mu ,\sigma ) \approx (1.235, 0.95)$ and normalized to the relevant amplitude. This underscores the challenge of identifying power-law behaviour in range-limited data, because although the log-normal distribution eventually diverges from the power law, it does so over a much larger domain than available in either Helium experiment (here or^7^). These fits scarcely differ in their goodness-of-fit criterion (the mean square error) and so offer no obvious way to reconcile the expected distribution with these divergent statistical conclusions.

### Findings from simulations

**Figure 3:**
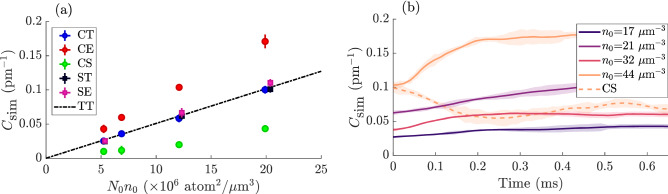
Simulations of release from the trap. (**a**) Steady-state values of the simulated contact. Simulations of condensates released from a cigar-shaped trap (CT) are consistent with the Tan theory (TT) before release, and show an increase in contact after the trap release (CE). A slow relaxation of the transverse trapping frequencies (CS) shows a decrease in line with the predicted value of the lower density. Spherical traps (ST,SE) lack any directions of tight confinement, wherein a longer interaction time prevents the escape of depleted particles as seen in cigar traps. (**b**) The time-dependence of the contact stabilizes after a time on the order of $1/\omega _x$, several hundred $\upmu$s. The expanded contact is consistently about 1.7 times the Tan theory. For comparison, the experimental control pulses are implemented after 2 ms of expansion. When the transverse trapping frequencies are slowly (1.2 ms) reduced by half (dotted line), the in-situ contact relaxes on a faster timescale than the ramp.

In order to understand whether the depletion could survive the expansion and to investigate what effects are taking place during the initial release, we performed simulations of the BEC expansion from harmonic traps using the first principles STAB method^33,44^. The simulations started from a cigar-shaped trap with parameters matched to the experimental conditions. The in-trap state before release from the trap at time $t=0$ (marked CT in Fig. 3a) was consistent with the adiabatic sweep theorem applied to the in-situ condensate. Following expansion from the cigar trap, the simulated tail amplitude increased and stabilized within a few hundred microseconds (CE in Fig. 3a), which is much slower than the timescale of the trap potential’s vanishing, and implies the far-field tails stabilize in appearance much sooner than the 2ms delay between the trap release and application of the RF and Stern–Gerlach pulses. Figure 3b shows the time evolution of the tail amplitude $C_{\mathrm{sim}}$ extracted from a $n(k)=C_{\mathrm{sim}}/k^4$ fit to the simulated density. In this configuration the steady-state value of the momentum tails was a factor of $C_{\mathrm{sim}}/{\mathscr {C}}=$1.64(9) above the predictions of Eq. (10). An analysis of the occupation of the tails according to (14), gives very similar factors ${\mathscr {A}}_{\mathrm{sim}}$ for the increase in the strength of the tails (relative to *in-situ* predictions) during evolution, as shown in Supplementary Table S2.

To understand the disagreement with earlier theory^23^, which predicted no depletion survival, we also investigated the effect of adiabatic expansion on the in-trap depletion. The characteristic healing timescale $t_{\xi }=\hbar /gn_0=15{-}40\,\upmu \hbox {s}$ in the centre of the trapped cloud is comparable to the trap release time $\tau _{\mathrm{release}}$, so a suspicion that adiabaticity is broken in the CE trap release simulations is warranted. For example, $t_{\xi }$ is a characteristic timescale for relaxation of density correlatons due to depletion after a quantum quench^61^. To test the hypothesis that the difference is due to our system breaking the adiabaticity assumed in Ref.^23^, we ran simulations in which the trap is not rapidly released, but ramped down to half transverse strength over a much longer time period (CS in Fig. 3). The in situ expression (Eq. 9) predicts that the depletion should reduce $\propto {\bar{\omega }}^{6/5}$ to about half its original value. Indeed it was found that the in-trap contact $C_{\mathrm{sim}}$ as well as the the tail strength $N_{k_{\mathrm{min}},k_{\mathrm{max}}}$ from (14) decreased roughly as predicted—see the dashed line in Fig. 3b and Supplementary Table S2, strongly supporting the hypothesis that adiabaticity is needed for agreement with the results of Ref.^23^.

As a check on whether we correctly identify the processes involved in depletion survival, we compared release of atoms from the experimental elongated clouds with spherically trapped clouds having the same central density $n_0$ and particle number *N*. These clouds are labelled (ST,SE) for initial and released clouds, respectively. We find that the survival of depleted atoms is reduced in the spherical trap compared to the elongated ones.

### Analysis of simulation results

Our understanding of the above dependencies in the simulations is that the survival and tail strength behaviour are a consequence of the rapid ramp-down of the trap and quench of density which allows escape of non-condensed particles, as well as their acceleration by the non-uniform mean-field energy of the condensate during the expansion.

In detail, after a quench into the untrapped regime, the condensate expands hydrodynamically on timescales of $1/\omega$, and the equilibrium depletion density drops in accordance with falling central density $n_0$ in Eq. (10). However, whether the actual density in k-space modes follows this equilibrium relationship depends on the reabsorption timescale. Low momentum depletion atoms are unable to escape the condensate before being reabsorbed and are absorbed back into the condensate in agreement with Ref.^23^. However, if reabsorption occurs slower than the change in density, the drop in depletion will be incomplete. High momentum atoms have sufficient velocity to escape the expanding cloud without being reabsorbed and thus transition to free atoms. In our system, as seen in Fig. S4 in the supplement, this concerns particles with wavenumber on the order of $k\gtrsim 2~\mu {{\text {m}}}^{-1}$, which in particular includes the high momentum tails that are the focus of the experiment. This is the same kind of escape mechanism seen for the appearance of halos of $k, -k$ paired atoms in supersonic BEC collision experiments^44,62,63^.

Moreover, an atom inside the BEC experiences an effective force from the gradient of the mean-field potential ${{\textbf {F}}} = -4\pi \hbar ^2 m^{-1}a \nabla n(x,t)$. This endows escaping depleted particles with a greater momentum. This phenomenon dubbed a “skiing effect”^64^ has been observed for the thermal part of the cloud in other experiments^65,66^, and for supersonic BEC collision halos^63,67,68^. For a scale-free distribution such as the $k^{-4}$ power law sought here, such a shift of momentum will manifest itself as an increase of the amplitude of the tails in the far-field, thus explaining how the observed depletion can appear stronger than in-situ. The simplest very rough estimate of this effect can be made by adding an energy of $gn_0$ to each atom during expansion, obtaining a modified density profile of the form $n(k)\rightarrow \approx {\mathscr {C}}k/(k^2-2gn_0m/\hbar ^2)^{5/2}$. This leads, for example, to a doubling of the apparent contact $C_{\mathrm{sim}}$ at $k\approx 6/\upmu$m for clouds with $n_0=39\,\upmu \mathrm{m}^{-3}$. Thus, this modification alone is not sufficient to explain the excess counts in the detection region.

A third element is that it is much easier for depletion atoms to escape and the acceleration is larger along the tightly-confined axes of a cigar-shaped cloud because the distances $R_{\perp }=(1/\omega _{y,z})\sqrt{2gn_0/m}$ are reduced by ${\bar{\omega }}/\omega _{y,z}$, whereas the initial mean depletion velocities in situ $v\sim \sqrt{2gn_0/m}$ are isotropic. Indeed, the simulations show that spherical clouds (SE) exhibit a much weaker effect than the elongated clouds (CE) in agreement with the longer escape time. This anisotropy effect also presents as an increase in $C_{{\text {sim}}}$ and ${\mathscr {A}}_{\mathrm{sim}}$ for simulation collection regions (ROI) that include a narrower range of angles around the tight trapping plane. Our ability to test this experimentally was limited because atoms with momenta larger than about 5 $\mu {{\text {m}}}^{-1}$ in the horizontal plane expanded beyond the detector’s active surface. Therefore, we obtain only weak evidence of such anisotropy in the experimental data, which is discussed in the Supplementary Material.

The above picture is corroborated by another observation within the simulations: During the expansion we observe a decrease in the total number of depleted particles (reabsorption) as seen in Supplementary Table S3 by comparing CE to CT and SE to ST values of $N_B$, and a simultaneous increase of the large-k population (forcing) described by $C_{\mathrm{sim}}$. A toy model of $k,-k$ depletion modes in a uniform gas undergoing external change in the background density was also investigated to verify our interpretation of the processes involved.

### Toy model of escape

The reabsorption mechanism and qualitative features of the escape of depletion from the condensate discussed above can be seen in a toy model of two *k* and $-k$ Bogoliubov modes in a uniform volume of gas at zero temperature when the background density is quenched due to external factors, as described in the supplementary material. The simplest such “caricature”, when the density $n_0$ is quenched to $n'<n_0$ at $t=0$ and then stays constant, has the occupation of each *k* mode evolve as

16 $$\begin{aligned} \rho (k,t) = \rho (k,0) - \frac{gn[\varepsilon _0(k)^2-\varepsilon (k)^2]}{4\varepsilon (k)^2\varepsilon _0(k)}\,[1-\cos 2\varepsilon (k) t]. \end{aligned}$$

**Figure 4:**
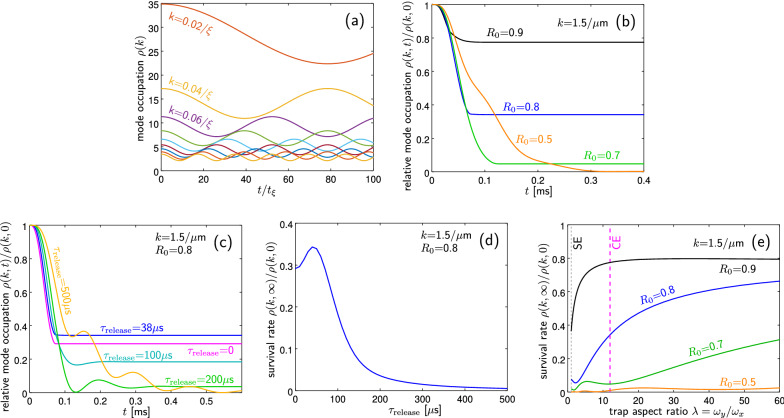
Toy model simulations for *k* and $-k$ modes in a uniform gas initially in the Bogoliubov ground state. (**a**) Behaviour after a “caricature” quench to half-density, as per Eq. (16) for modes with $k\xi =0.02,0.04,\dots ,0.18$. The remaining panels concern the better toy model of Eqs. (S10, S17) with parameters like the full simulation with $\omega =902 \times 895 \times 71$ Hz, $N=455852$, $n_0=43.66/\upmu \text {m}^3$ peak density, and $\tau _{\mathrm{release}}=38\,\upmu \hbox {s}$, and show the mode occupation evolution relative to the initial value (the survival rate). (**b**) For different initial locations in the condensate in the narrow direction: $R_0=y/R_{\perp }$ where $R_{\perp }=(1/\omega _y)\sqrt{2gn_0/m}$, and initial $k=1.5/\upmu \hbox {m}$. (**c**) Evolution of the relative occupation for different ramp speeds $\tau _{\mathrm{release}}$. Cycles of reabsorption are seen for the slow ramps. (**d**) Final survival rate for the same parameters. (**e**) Shows the dependence of the final survival rate on the trap aspect ratio $\lambda$, when initial central density $n_0$ is kept constant. The magenta dashed line indicates the experimental $\lambda =12$ (like CE simulations), the black dashed line a spherical trap $\lambda =1$ (like SE simulations).

Here, $\varepsilon _0(k)$ and $\varepsilon (k)$ are given by (3) using the initial $n_0$ and later *n* values of density, respectively. The reabsorption comes about then via the initial dip of the Rabi oscillations seen in Fig. 4a. The Rabi oscillations are between the two superpositions corresponding to the initial Bogoliubov ground state and the final one. However, in the actual expansion it can happen that later (return) stages of the Rabi oscillations never eventuate if the density drops faster than the oscillation frequency; roughly $\omega _{\perp }\gtrsim 2\varepsilon (k)$.

Figures 4b–e show the behaviour of a more careful toy model given by Eqs. (S10) and (S17) (requiring numerical integration) in which the background density decays in a time-dependent fashion that at least qualitatively approximates a significant part of what happens during release. Panel (b) concerns escape of particles that start in the outer parts of the cloud ($R_0=y/R_{\perp }\gtrsim 0.5)$. Panels (c,d) show the dependence of survival on the speed of the ramp which turns off the trap, indicating that ramp speeds $\tau _{\mathrm{release}}\lesssim 100\,\upmu \hbox {s}$ are mostly neutral for the effect but slower ramps strongly suppress the escape. Panel (e) considers the survival rate for different trap aspect ratios, including the case of $\lambda =12$ like in the experiment (CE) and the spherical case (SE). Survival is greatly aided by elongated traps.

There are still many effects missing from the toy model compared to the STAB simulations (high momentum atoms on trapped trajectories temporarily outside the condensate at time of release, multidirectional flight of the depleted atoms, reduced skiing due to simultaneous collapse of the condensate density, energy-momentum uncertainty, the effect of the remnant trapping potential on depletion atoms, to name a few) and survival rate is lower than in the full 3D simulations. It does, though, give a first qualitative underpinning for the escape effects seen in the full many-mode simulations.

## Discussion

We find that the number of atoms in the large-*k* tails in the far-field is consistent with the tail amplitude scaling as a linear function of the product $N_0n_0\propto (N_{0}^7{\bar{\omega }}^6)^{1/5}$, in line with Tan’s theory of the contact (Eqs. 10, 14). Neither thermal populations nor a number of technical effects (imaging, single particle processes, background noise) can be expected to have the same $N_0n_0$ scaling. However, the effect size is significantly different than naively expected from in-situ values by a factor of order 8(3), and from simulated values by a factor of 5(3) which is not accounted for by any obvious systematic effects.

The earlier experiment by Chang et al.^7^ also noted the $N_0n_0$ scaling and an excess by a factor of about 6 that falls within our error bars. However, recent investigations^35^ found that this was correlated with the presence of a small impurity fraction consisting of $m_{j}=0$ atoms ($\sim \,1$%) in their optically trapped cloud, which was otherwise spin polarised in the $m_{j}=1$ state. When the impurity fraction was reduced to 0.05% tail survival was no longer observed. In contrast, our experiment is conducted in a magnetic trap, which is unable to confine any spin state other than the $m_{j}=1$ state. Thus our results can not be explained by the presence of similiar trapped impurities.

While the thermal quasiparticles in the Bogoliubov picture simply map onto the thermal population of constituent particles of the same momentum (see, for example^45^, Chap. 8.3. or Ref.^2^), a remnant thermal population is not a good candidate to explain the observations. This is because it decays super-exponentially with *k* (Bose–Einstein distribution), and hence does not account for the atoms we observe beyond $k\gtrsim 6~\mu {{\text {m}}}^{-1}$, even though it is subject to the same mean-field forcing as the depletion^65^. We can show this with a simple calculation, noting that on physical grounds the maximum energy that can be imparted by the “skiing effect”^64^ is $\mu =gn_0$, where $n_0$ is the initial density in the centre of the cloud. For an atom with momentum $k=6\,\upmu {{\text {m}}}^{-1}$, at the edge of the thermal region in the densest cloud we consider (44 $\upmu {{\text {m}}}^{-3}$), the additional energy $\mu$ imparts at most a momentum shift of order 0.7 $\upmu {{\text {m}}}^{-1}$, which is insufficient to account for the population detected as far out as $k=10\,\upmu {{\text {m}}}^{-1}$. The phonon/particle changeover is also not responsible for the inflections seen at high *k* in Fig. 1 because this changeover occurs at $k\sim \,1/\xi \approx 2\,\upmu \mathrm{m}^{-1}$.

Another candidate explanation to consider would be large depletion produced after release in the short-lived mixed-species condensate (where the $m_J=0,1$, and $-1$ clouds overlap after the Landau–Zener sweep). This could have the observed $N_0n_0$ scaling. However, the expression for the contact in a mixed-species bosonic gas^30^ can be combined with the energy of a condensed mixture^45^ to show that the contact in spin-mixed systems is bounded from above by the contact of the same system polarized in the most strongly-interacting state^25^. The inter-spin scattering lengths $a_{ij}$ (between He$^*~$atoms in the *i* and *j* spin states) are not fully characterized by experiments, but can be estimated to be $a_{11}=a_{-1-1}=a_{01}=a_{0-1}\approx 140~a_0$, $a_{00}=120~a_0$ and $a_{1-1}\approx 60~a_0$, in terms of the Bohr radius $a_0$^69^. Thus, the contact in a He$^*~$condensate is maximized when the cloud is purely polarized in the $m_J=1$ state. Any mixture of He${}^*$ spin states is thus predicted to have a lower contact (and thus less-populated tails) than the initial condensate, which appears to rule out this route to explain our observations.

On the other hand the simulations and toy model demonstrate a route for escape of the fast depletion atoms from the cloud, and indicate that the survival of the quantum depletion into the far-field is possible when the release is non adiabatic, but not as a straightforward mapping into the far-field density. The latter is due to the dispersal of the mean-field energy into kinetic energy, which imparts some acceleration to the atoms during the early stages of the expansion^65^

We thus are led to surmise that the experimentally observed tails are indeed a remnant of the quantum depletion (per the observed scaling with $N_0n_0$ and $\approx k^{-4}$ that matches the Tan theory, qualitative similarity in behaviour to the simulations, and lack of convincing counter-hypotheses), albeit subject to some physical effect during the expansion or some nonequilibrium enhancement in the trapped state.

In conclusion, we find statistically robust evidence that the quantum depletion can, remarkably, survive the expansion and dilution of its original condensate under certain conditions. Our simulations also demonstrate a mechanism by which the uncondensed quantum depleted atoms of a single species can be visible in the far-field momentum distribution, and that the hydrodynamic approximation does not capture sufficient short-wavelength information to make detailed predictions about the high-momentum behaviour. We thus find a partial explanation for the experimental deviation of the far-field distribution from both the in-situ and the hydrodynamic pictures, although there is an unexplained discrepancy at this time between theory and experiment as to the amount of this growth. The results reported here expand the growing body of data and knowledge regarding the somewhat mysterious behaviour of the far-field quantum depletion^7,13,23,35^.

Since the exact mechanism responsible for the impurity effects seen in Ref.^35^ remains unrecognised, it is uncertain whether it can also be responsible for our measurements of tail strength well in excess of the single-species simulation. Our experiment does not involve impurities in the initial trapped cloud.

## Methods

### Experimental setup

**Figure 5:**
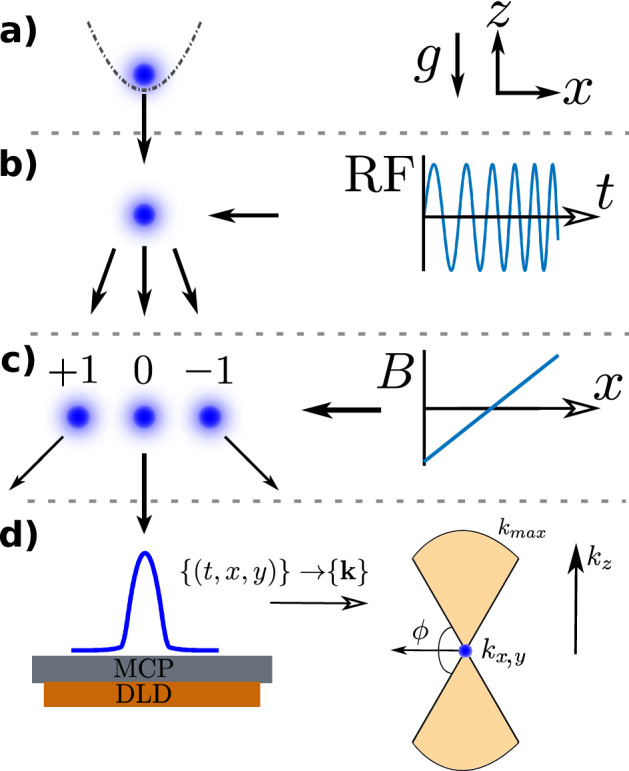
Sketch of the experimental sequence. A BEC is released from a harmonic trap (**a**) and expands during freefall before being split into a superposition of the $m_J\in \{-1,0,1\}$ states (**b**) by an RF chirp. A magnetic field gradient separates the clouds (**c**) ensuring that only the magnetically insentitive $m_J=0$ cloud lands on the detector (**d**), from which the momentum information is reconstructed. Due to the finite detector radius, the collection region in momentum-space is restricted to two vertically-oriented spherical segments (shaded region) whose boundary subtends an angle ${\phi _c}=\pi /3$ with the horizontal (*x*, *y*) plane. The quantum depletion lies in the dilute tails at large momentum $\gtrsim 6\mu {{\text {m}}}^{-1}$ (see Fig. 1).

Our experimental sequence for measurement runs as described in the “Experimental meaurements” section above is shown schematically in Fig. 5.

We prepared our BECs via forced evaporative cooling in a harmonic magnetic trap with trap frequencies $\approx (45,425,425)$ Hz and a DC bias stabilized by our auxiliary field compensation coils^47,70^. For the tight trap we increased the coil current after the cooling sequence to obtain trapping frequencies $\approx \,(71,902,895)$ Hz, ramping the current as a sigmoid step function to minimize in-trap oscillations. Note that the weak (*x*) axis of the trap is horizontal, with tight vertical confinement. The RF pulse was created by a function generator, amplified, and applied to the experiment chamber by a coiled antenna inserted into the BiQUIC coil housing. The pulse swept from 1.6 to 2.6 MHz over 1ms and was centred on the resonance between the $m_J$ states. The determination of the transfer efficiencies $\eta _J$ for each of the $m_J$ states is discussed below. The sweep was $10^6$-fold wider than the RF width of the BEC to ensure uniform transfer at all momenta. Immediately after the RF sweep, the bias coils are switched off and auxiliary push coils in the vertical (Z) and weak horizontal (X) axes are activated using a fast MOSFET switch to implement a Stern–Gerlach deflection of the $m_J = -1,$ and $+1$ atoms, such that only $m_J=0$ state atoms reach the detector. The Stern–Gerlach (SG) pulse was designed by increasing the pulse duration until the $m_j=\pm 1$ clouds were given sufficient velocity to reach the edges of the detector ($\approx 10$ cm/s), and then doubling the current passed through the field-generating coils.

We use an 80mm diameter multichannel plate and delay-line detector stack^49^ located 848 mm below the trap, which registers the arrival times and positions (*t*, *x*, *y*) of each atom. The velocity of each atom relative to the centre of mass of each cloud is calculated by $(v_x,v_y,v_z) = t_{i}^{-1}(x_i-{\bar{x}},y_i-{\bar{y}},\tfrac{1}{2}g_0(t_{cen}^2-t_{i}^{2}))$, where $g_0$ is the local gravitational acceleration, the overbar denotes the within-shot average and $t_{cen}$ is the time of flight of the centre of mass of the cloud. The far-field momentum is thus obtained via $m{{\textbf {v}}} = \hbar {{\textbf {k}}}$, noting that this cannot be identified with the in-situ momentum (see “Discussion” section). The space and time resolution of the detector are $100\,\upmu \hbox {m}$ and $3\,\upmu \hbox {s}$, respectively^71^. Sets of ten experimental runs were interleaved with calibration measurements to determine the shot-to-shot variation in atom number, trapping frequencies, magnetic state transfer efficiency, and noise contributions in the manner described in the supplementary material.

The detector quantum efficiency (QE) of ${\eta _Q=}8(2)\%$ was determined from analysis of the squeezing parameter of correlated atoms on the opposite sides of scattering halos^72–74^. A second factor affecting the total collection efficiency $\varepsilon$ is that the *k*-space field of view is restricted by the detector radius to $k\lesssim \,5 /\upmu \mathrm{m}$ in the (*x*, *y*) plane, which is only just sufficient to reach past the edge of the thermal region. We thus face a tradeoff in the choice of $k_{{\text {max}}}$, therefore we define the bounds of our region of interest (ROI) by the minimum elevation angle $\phi _c=\pi /3$ rad above the (*x*, *y*) plane and an upper bound of $k_{{\text {max}}} = 10\,\mu {{\text {m}}}^{-1}$ (beyond which the signal to noise ratio becomes too poor). This amounts to an ROI consisting of two vertically oriented spherical segments, each with half-angle $\pi /6$ from the *z* axis, encompassing a total solid angle of ${\Omega _{ROI}=4\pi (1-\sin {\phi _c})=}0.13 \times 4\pi$ steradians.

We must also account for the state-transfer efficiency of ${\eta _0=}25(2)$% during the RF sweep, and combine all these factors into the total efficiency $\varepsilon {=\eta _Q\eta _0(1-\sin {\phi _c})}\approx \,0.23(5)\%$. The dominant uncertainty in the collection efficiency $\varepsilon$ is the 25% error in the detector quantum efficiency (QE), whereas the other factors (cutoff angle $\phi _c$ and transfer efficiency $\eta _0$) are more precisely known.

We performed the analysis of the depletion tails described above for a range of $\phi _c$ and values of the QE $\eta$ and found that the excess of counts (expressed as $\Lambda _{{\text {fit}}}/\Lambda _{{\text {pred}}}$) was not significantly affected. This is summarized Supplementary Table S1.

### Simulations

The STAB method (stochastic Time-Adaptive Bogoliubov)^33,44^ uses the positive-P representation^75,76^ to describe Bogoliubov quasiparticles around a dynamically evolving condensate^32^. This allows for straightforward treatment of inhomogenous and evolving condensates with their associated quantum depletion, without the need to diagonalise the Bogoliubov-de Gennes equations. The systems considered here require $4{-}6 \times 10^6$ modes for simulation, so avoiding diagonalisation is very relevant. Previous use of the STAB method^33,44,63,67,74,77–79^ has been according to the equations described in detail in Ref.^33^ which relied on a separation of the condensate and Bogoliubov quasiparticles in k-space that arose from initial conditions and system dynamics. Here this does not occur, and there is a significant overlap in momentum space. The standard STAB formulation leads to an unphysical amplification of the part of the Bogoliubov field that overlaps with the condensate. Therefore a theory that explicitly imposes orthogonality between condensate and Bogoliubov modes is required. We summarise our approach here, with some technical details in the supplemental material. Details of the derivation and proper benchmarking of the modified method will be reported in Ref.^80^.

#### Orthogonalised STAB method

In terms of operators, the Bose field of the atoms $\widehat{\Psi }({\mathbf {x}},t)$ is written as

17 $$\begin{aligned} \widehat{\Psi }({\mathbf {x}},t) = \phi ({\mathbf {x}},t) + \widehat{\Psi }_B({\mathbf {x}},t), \end{aligned}$$

where $\phi ({\mathbf {x}},t)$ is the condensate order parameter described in 3-dimensional space ${\mathbf {x}}$, and $\widehat{\Psi }_B({\mathbf {x}},t)$ is a relatively small operator fluctuation field. The smallness requirement can be written

18 $$\begin{aligned} N&=\int d^3{\mathbf {x}}\ |\phi ({\mathbf {x}},t)|^2, \end{aligned}$$

19 $$\begin{aligned} N&\gg \int d^3{\mathbf {x}}\left\langle \widehat{\Psi }^{\dagger }_B({\mathbf {x}},t)\widehat{\Psi }_B({\mathbf {x}},t) \right\rangle =N_B=N\delta _B, \end{aligned}$$

i.e. $N_B$ the number of particles in the Bogoliubov field is small overall, but locally the Bogoliubov field density need not be smaller then the condensate—$\delta _B$ is the small parameter of the theory^81^. The condition (19) allows one to discard third and higher orders of $\widehat{\Psi }_B$ in the effective Hamiltonian (the Bogoliubov approximation). A second condition, not applied in standard STAB, but present in more precise flavours of Bogoliubov theory is

20 $$\begin{aligned} \int d^3{\mathbf {x}}\ \widehat{\Psi }^{\dagger }_B({\mathbf {x}},t)\phi ({\mathbf {x}},t)= 0, \end{aligned}$$

which imposes orthogonality and prevents seeping of condensate atoms into the fluctuation field $\widehat{\Psi }_B({\mathbf {x}},t)$.

The condensate order parameter $\phi ({\mathbf {x}},t)$ is assumed to evolve according to the Gross–Pitaevskii equation (correct to leading order, given (19)):

21 $$\begin{aligned} i\hbar \frac{d\phi }{dt} = \left[ -\frac{\hbar ^2}{2m}\nabla ^2+g|\phi |^2 +V({\mathbf {x}},t)\right] \phi , \end{aligned}$$

and is normalised to the (conserved) total number of particles $\int d^3{\mathbf {x}}\ |\phi ({\mathbf {x}},t)|^3=N$. The $g=4\pi \hbar ^2a_{1,1}/m$ is the s-wave contact interaction between He${}^*$ atoms in the initial $m_J=1$ state (we take $a_{1,1}=7.51$nm), and $V({\mathbf {x}},t)$ is the trap potential with in general time-dependent frequency. We then represent the Bogoliubov quasiparticles using the positive-P representation^33,75^, which leads to the following equations of motion:

22 $$\begin{aligned} i\hbar \frac{d\psi _B}{dt}= & {} \left[ -\frac{\hbar ^2}{2m}\nabla ^2+g|\phi |^2+V({\mathbf {x}},t)\right] \psi _B +{\mathscr {P}}_{\perp }\left\{ g|\phi |^2\psi _B + g\phi ^2{\widetilde{\psi }}_B^* + \sqrt{-ig}\ \phi \,\xi ({\mathbf {x}},t) \right\}, \end{aligned}$$

23 $$\begin{aligned} i\hbar \frac{d{\widetilde{\psi }}_B}{dt}= & {} \left[ -\frac{\hbar ^2}{2m}\nabla ^2+g|\phi |^2+V({\mathbf {x}},t)\right] {\widetilde{\psi }}_B +{\mathscr {P}}_{\perp }\left\{ g|\phi |^2{\widetilde{\psi }}_B + g\phi ^2\psi _B^* + \sqrt{-ig}\ \phi \,{\widetilde{\xi }}({\mathbf {x}},t) \right\}. \end{aligned}$$

Here the ket $\psi _B({\mathbf {x}},t)$ and bra ${\widetilde{\psi }}_B({\mathbf {x}},t)$ amplitudes provide the positive-P representation of the Bogoliubov field ${\hat{\Psi }}_B({\mathbf {x}},t)$ in 3D space. We used the robust stochastic integration procedure described in Ref.^82^. The $\xi ({\mathbf {x}},t)$ and ${\widetilde{\xi }}({\mathbf {x}},t)$ are independent white Gaussian noise fields of zero mean and variance:

24 $$\begin{aligned} \langle \xi ({\mathbf {x}},t)\xi ({\mathbf {x}}',t')\rangle = \langle {\widetilde{\xi }}({\mathbf {x}},t){\widetilde{\xi }}({\mathbf {x}}',t')\rangle = \delta ^3({\mathbf {x}}-{\mathbf {x}}')\delta (t-t'). \end{aligned}$$

An ensemble of field trajectories with independent noise in each trajectory and in each trajectory’s initial state is generated to represent the Bogoliubov field. We typically used ${\mathscr {S}}=4000$ trajectories. Notably, the Eqs. (22) and (23) allow not only for production of additional Bogoliubov quasiparticles quantum depleted from the condensate but also for their reabsorption. The main additional element in (22) and (23) compared to the standard STAB equations^79^ is the projection ${\mathscr {P}}_{\perp }$ which imposes the orthogonality requirement (20) and avoids the aforementioned amplification of the Bogoliubov field where it overlaps with the condensate. The projection ${\mathscr {P}}_{\perp }$ of a field $f({\mathbf {x}})$ can be carried out efficiently by

25 $$\begin{aligned} {\mathscr {P}}_{\perp } f({\mathbf {x}}) = f({\mathbf {x}})- \frac{1}{N}\left[ \int d^3{{\mathbf {x}}'}\ \phi ({{\mathbf {x}}'})^*f({{\mathbf {x}}'})\right] \,\phi ({\mathbf {x}}). \end{aligned}$$

The kinetic part of the evolution Eqs. (21)–(23) is also carried out efficiently by a split-step approach which evaluates kinetic terms in k-space and the rest in x-space, moving between k-space and x-space using a fast Fourier transform. Calculation of observables is described in the Supplementary Material.

#### Initial condition

Our simulations aim to study the evolution of the quantum depletion particles in $\widehat{\Psi }_B$ after release from the trap. We use a zero temperature initial condition, since the object is to study the behaviour of the high momentum tails beyond the edge of the thermal cloud, in which $T>0$ effects are negligible. The $T=0$ initial state is also more straightforward to obtain, allowing one to use lower k-values to access the $k^{-4}$ tails, since they are not obscured by the stronger thermal cloud at intermediate momenta. This significantly reduces the size of the computational lattice needed. For the low temperatures in the experiment we do not expect any significant interaction between the behavior of the thermal cloud and the depleted atoms because both are well approximated by the Bogoliubov approximation which neglects interactions between excited modes. Therefore the neglect of the thermal cloud does not significantly affect the properties of the higher k depletion or its evolution.

However, one cannot use the standard Gross–Pitaevskii ground state since that has 100% condensate and no quantum depletion. The task of generating a cloud with the appropriate depletion in such a large nonuniform system turns out to be nontrivial. Conceptually the issue is simple—diagonalise the Bogoliubov Hamiltonian, and give the well known Bogoliubov $T=0$ occupation to each quasiparticle mode. However, for a system with $10^6$ modes diagonalisation is not a good option. Our solution to this situation is to make a calibrated quantum quench from the Gross-Pitaevskii solution to the full Bogoliubov equations of motion, which provides a state with an appropriate quantity of quantum depletion. The technique is described in detail in the Supplementary Material.

#### Simulation types

Several types of simulations were made, with shorthand labels as per Fig. 3, and summarised in Supplementary Table S3:

*(CE)* Release of atoms from the trap, as in the experiment. Here the potential was reduced exponentially

26 $$\begin{aligned} V({\mathbf {x}}) = \frac{m}{2}\left( \omega _x^2x^2+\omega _y^2y^2 +\omega _z^2z^2\right) \,e^{-t/\tau _{\mathrm{release}}}, \end{aligned}$$

with time constant $\tau _{\mathrm{release}}=37.5\,\upmu$s, matched to the experiment. The initial trap frequencies were $\omega =425 \times 425 \times 45$ Hz and $\omega =902 \times 895 \times 71$ Hz, and two variants of the initial state were simulated: a low density and a high density cloud.

##### (CS)

Slow decrease of the transverse trapping frequencies by a factor of two. Here we ramped the trap as follows:

27 $$\begin{aligned} V({\mathbf {x}}) = \frac{m}{2}\left[ \left( \omega _x^2x^2+\omega _y^2y^2\right) \left( 1-\frac{t}{2t_{\mathrm{ramp}}}\right) ^2 +\omega _z^2z^2\right] , \end{aligned}$$

with timescales of order 1-2ms (see Supplementary Table S3). The simulations were run up till $t=t_{\mathrm{ramp}}$ when the transverse trap frequency was half the original one.

##### (ST,SE)

Trap release of spherical clouds. The velocity distribution of the depletion atoms is isotropic in situ, being given by $mv^2/2 \approx gn_0$. However, the distance to travel to escape reabsorption depends on the cloud shape. In particular, escape is made easier in the tight trap directions (less distance to travel), and harder in the long trap direction. Here we used spherically trapped clouds having the same central density $n_0$ and particle number *N*. These clouds had isotropic trapping frequency ${\overline{\omega }}=(\omega _x\omega _y\omega _z)^{1/3}$ and are labelled (ST). Trap release (SE) followed (26) as before.

## Supplementary Information

Supplementary Information.
