# Supplementary material for: On the survival of the quantum depletion of a condensate after release from a magnetic trap
Source: Sci Rep. 2022 Aug 1;12:13178. doi: 10.1038/s41598-022-16477-9 (PMC9343431; doi:10.1038/s41598-022-16477-9)
Supplement: Supplementary file 1 — Supplementary Information. [file 41598_2022_16477_MOESM1_ESM.pdf]

Supplementary information to
On the survival of the quantum depletion of a condensate after release from a magnetic trap

J. A. Ross, P. Deuar, D. K. Shin, K. F. Thomas, B. M. Henson, S. S. Hodgman, and A. G. Truscott*
* Corresponding author email: andrew.truscott@anu.edu.au

Experimental details

Peak density calibration

The quantum depletion and contact are both predicted to depend solely on the condensed number and trapping frequencies via the condensate density, hence it is important to determine both quantities accurately.

The sole experimental parameters in the expression for the peak density (Eqn. (11))are the geometric trap frequency $\bar{\omega} = (\omega_x \cdot \omega_y \cdot \omega_z)^{1/3}$, and N_0, the number of atoms in the condensate. We simultaneously determine the total atom number N and trap frequency $\bar{\omega}$ in a single shot using a pulsed atom laser and use the thermal fraction η_T (see below) to determine the condensed number $N_0 = (1 - \eta_T)N$.

The pulsed atom laser consists of a series of Fourier-broadened RF pulses centred on the minimum Zeeman splitting in the trap. The pulse transfers atoms in the trap to the untrapped $m_J = 0$ state with an approximately constant transfer rate across the cloud[49,71]. We outcouple approximately 2% of the atoms per 100 μs pulse for ≈200 pulses, which eventually depletes the entire trap. The atom laser thus prevents the detector from saturating and allows an accurate determination of the atom number, up to a factor of the quantum efficiency. We determine the trapping frequencies by inducing centre-of-mass oscillations with a magnetic impulse, and find the oscillation period from the atom laser pulses[83].

Determining spin transfer efficiency

To calibrate the transfer efficiencies, we applied a weaker Stern-Gerlach than for the depletion measurement, such that each m_J cloud hits detector at different times (and positions), as illustrated in Figure S1. The efficiencies η_J cannot be calculated by counting the atoms in each cloud because the detector saturates during the peak condensate flux. However, we can compare the thermal parts. We align each cloud along the time (Z) axis and compute the pointwise fraction of the atomic flux $\phi(t)$ accounted for by each cloud, $\eta_j(t) = \phi_j(t)/\sum_j \phi_j(t)$, as depicted in Figure S1 (a-c). The ratio of densities between the clouds is roughly constant in the thermal part (Figure S1 (c)), indicating the absence of saturation effects in the thermal part and a spin transfer efficiency that is independent of k. The fraction of the original cloud transferred into each m_J state is determined by taking the average $\langle \eta_j(t) \rangle$ over the thermal tails.

We find these efficiencies are approximately 74%, 24%, and 2% in all runs for the $m_J = +1$, 0, and -1 states, respectively. While the $m_J = 0$ and $m_J = 1$ clouds clearly saturate the detector, the small fraction (≈ 2%) of the atoms transferred to the $m_J = -1$ state does not (Figure S1 (d)). A bimodal fit to the condensed and thermal parts, plus constant background, yields the thermal fraction η_T and condensed fraction $1 - \eta_T$.

Noise sources

In early tests of our measurement sequence we noticed a contamination of the signal by spurious counts. Specifically, an extremely dilute remnant of the $m_J = +1$ cloud was observed as a small peak in counts in the region $-10\mu\mathrm{m}^{-1} \lesssim k_z \lesssim -8.5\mu\mathrm{m}^{-1}$. We inferred these were remnant counts from the $m_J = +1$ cloud as they were still visible when we ran an experimental sequence without the Landau-Zener transfer, but including the Stern-Gerlach pulse.

While the cause of the appearance of the remnant counts is unclear, we observe that the count density outside the region of interest is similar in both the shots with the RF pulse (Landau-Zener transfer) and those without. We note that only about one in a million atoms from the $m_J = 1$ cloud are present in this manner in a given shot. On average, this accounted for approximately $N_{mJ=+1} = 1$ atom per shot

when running the sequence without the Landau-Zener transfer, and approximately half this amplitude after applying the pulse. As the signal of interest was not the angular density profile but the integrated number of counts, we corrected for this contribution by subtraction, $N_{k_{\min},k_{\max}} = (N^{\text{detected}}_{k_{\min},k_{\max}} - \eta_1 N_{m_J=+1})/(\eta_0 \times \eta_Q \times \Omega_{\text{ROI}})$, where Ω_{ROI} is the fraction of the sphere retained by the windowing procedure described in the main text. and η_Q is the detector quantum efficiency. Such counts constitute about 10(5)% of the detection events in the ROI and are not sufficient to account for the excess tail strength. We hypothesize that the remnant counts are atoms transferred into the $m_J = 0$ state by non-ideal behaviour of the Stern-Gerlach pulses or magnetic field switches.

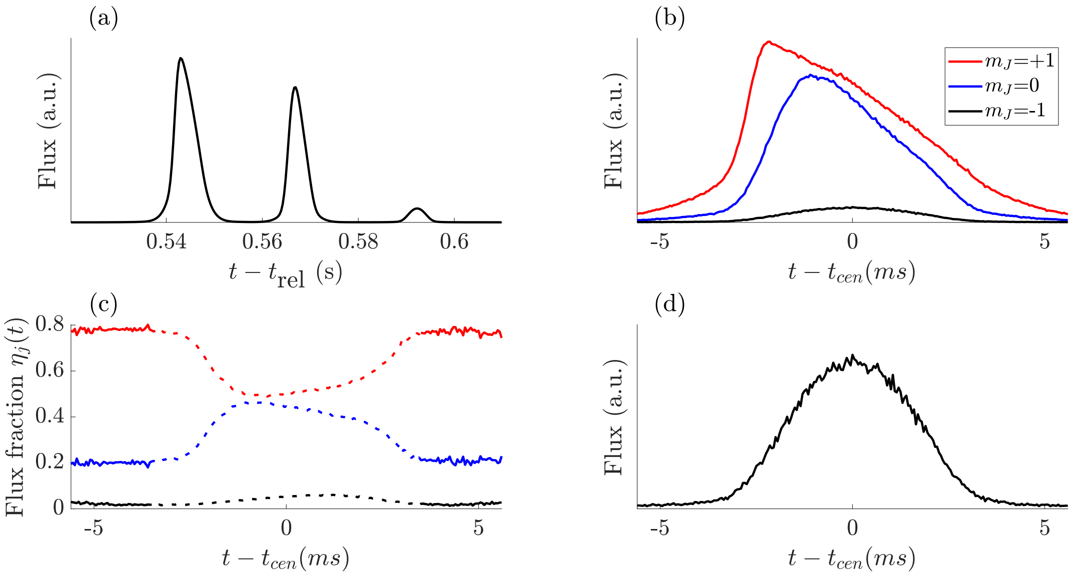

Figure S1. Determining the RF transfer efficiency. The time-of-flight profiles of each pulse are resolved (a) by applying a weak Stern-Gerlach pulse during the time of flight. The pulses are aligned with respect to their centre-of-mass (b) and used to determine the pointwise fraction ((c), dotted line). Detector saturation is evident in the peaks (dashed lines), but not in the thermal tails (solid lines), which are used to compute the transfer efficiency. Because of its lower flux, the $m_J = -1$ pulse does not show any clear evidence of saturation (d) and is used to determine the thermal fraction and hence N_0.

Dependence of tail properties on the ROI

Table S1 shows the absolute and relative tail amplitude estimates Λ_{fit} and $\Lambda_{\mathrm{fit}}/\Lambda_{\mathrm{pred}}$, respectively, that proceed from variations of the ROI and assumed quantum efficiency in the analysis of the experimental data. It is seen that neither the uncertainty in the collection efficiency nor the choice of elevation angle cutoff ϕ_c have a significant effect on the findings.

QE	fit r^2	Λ_{fit}	$\Lambda_{\mathrm{fit}}/\Lambda_{\mathrm{pred}}$
0.05	0.83	0.2(0.1,0.3)	6.8(4.5,9.1)
0.06	0.83	0.3(0.2,0.4)	7.4(4.9,9.8)
0.07	0.83	0.3(0.2,0.4)	7.8(5.2,10.5)
0.08	0.83	0.4(0.3,0.5)	8.3(5.5,11)
0.09	0.83	0.5(0.3,0.6)	8.7(5.8,11.6)
0.1	0.83	0.6(0.4,0.7)	9.0(6.0,12.1)
0.11	0.83	0.6(0.4,0.8)	9.4(6.2,12.5)
ϕ_c	fit r^2	Λ_{fit}	$\Lambda_{\mathrm{fit}}/\Lambda_{\mathrm{pred}}$
80°	0.82	0.1(0.0,0.1)	9.3(6.0,12.7)
70°	0.86	0.2(0.1,0.3)	9.2(6.4,12)
60°	0.83	0.4(0.3,0.5)	8.3(5.5,11)

Table S1. Tail predictions with modified ROI or assumptions about the value of quantum efficiency (QE). A change in quantum efficiency (QE) presents by changing the value of ε used in the prediction and also through the factor of $N_0^{7/5}$ used to compute the condensate density n_0. These effects partially cancel to produce a weak scaling of $\Lambda_{\mathrm{fit}}/\Lambda_{\mathrm{pred}}$ with respect to ε. Re-running the analysis using different QE and the standard ROI with $\phi_c = \pi/3$ yields fits that barely differ in the goodness-of-fit criterion and present comparable results for Λ_{fit}. We also find that the choice of collection area defined by the elevation angle cutoff ϕ_c (while using the best QE value of 0.08) has a weak effect on the result, but below statistical significance. Terms in brackets are the upper and lower 95% confidence intervals. Neither the uncertainty in the collection efficiency nor the choice of elevation angle cutoff ϕ_c have a significant effect on the findings.

Statistical details

In Figure S2 we illustrate how fixing the scaling exponent α when fitting to a given dataset obscures the enormous covariance between α and the scale coefficient C_α. The red diamonds in Figure S2 show the fit exponent and amplitude obtained from a fit of Eqn. (13) to single data sets, each representing a different BEC density. The variation in the fit exponent is small (mean 4.2, standard deviation 0.4), and although the mean α from the fits is only half a standard deviation (one standard-error interval) different from 4, the corresponding difference in amplitude C varies exponentially with α, spanning over six orders of magnitude.

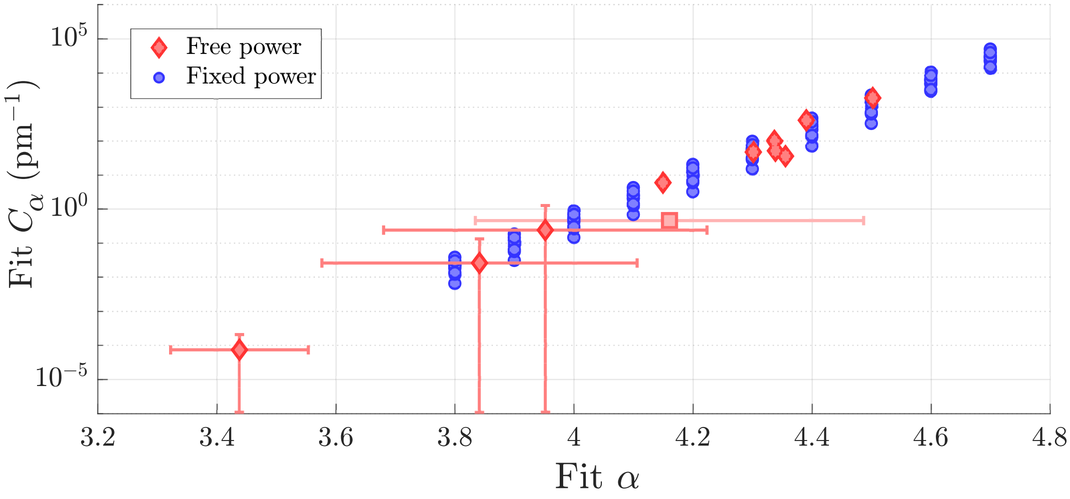

Figure S2. Illustrating the large systematic errors in a fit to the density using a power law ansatz. If the fit to the density profile (Fig 1 in the main text)) is replaced by Eqn. (13) (also in main) and α and C_α used as fit parameters, it gives a wide variation in best-fit exponents and scale coefficients between each data set (red diamonds). The red square shows the mean fitted α and (geometric) mean C_α with standard deviation (in α) shown as error bars. Blue circles show the amplitude coefficient C_α obtained from the same data sets with α fixed at a number of values within the range of the free fit results. The choice of α strongly determines the coefficient C_α, but the error bars (standard errors in fit parameters) are smaller than the markers in all cases.

The blue circles in Figure S2 show the amplitudes returned from fits to data from all datasets when α is constrained to the set values as shown. These set values are chosen within about one standard deviation of the mean α from the two-parameter fits to separate datasets. These blue fits scarcely differ in their goodness-of-fit criterion (the mean square error) and so offer no obvious way to reconcile the expected distribution with these divergent statistical conclusions. Furthermore, this is not reflected in the error estimates in the fitting routines: The error bars representing the uncertainty in parameters from the fixed-α fits are smaller than the markers used in Figure S2. A linear fit reveals that $d\log_{10} C_\alpha / d\alpha \approx 6.8$.

Ultimately, the question of whether the data is drawn from a power law at all is not amenable to a decisive conclusion. For example, a log-normal distribution can produce similarly accurate predictions to the power law (see Figure 2 in the article) although there is no physical hypothesis that predicts such a distribution. This points to one of the primary challenges with power laws; the exponents are strongly entwined with the rate of occurrence of rare events, which by definition are subject to large statistical fluctuations and and thus subvert even the most meticulous investigations. These problems with fitting power laws are ubiquitous, and made more difficult by the small range of k which are visible in the helium experiments. In general, estimating the exponent of a purported power law is difficult and requires data spanning several orders of magnitude in scale[59,60,84,85], which are not present in either of the helium experiments to date.

Nevertheless — the analysis of Fig. S2 does allow us to at least say that the data is inconsistent with power laws outside the range $3.3 < \alpha < 4.7$.

Simulations

Observables

Detailed data extracted from the simulations are given in Table. S3.

The total quantum depletion of the condensate δ_B is given by

$$\delta_B = \frac{N_B}{N} = \frac{1}{N}\int d^3\mathbf{x}\,\langle \mathrm{Re}\left[\psi_B(\mathbf{x},t)\,\widetilde{\psi}_B^*(\mathbf{x},t)\right]\rangle_{\text{stoch.}} \tag{S1}$$

Stochastic averaging over all trajectories in the ensemble is denoted by $\langle\cdot\rangle_{\text{stoch.}}$. The density of the depleted particles is evaluated by the standard positive-P expression,

$$n_B(\mathbf{k}) = \left\langle \widehat{\Psi}_B^\dagger(\mathbf{k},t)\widehat{\Psi}_B(\mathbf{k},t) \right\rangle = \text{Re}\,\langle \widetilde{\psi}_B^*(\mathbf{k},t)\psi_B(\mathbf{k},t)\rangle_{\text{stoch.}}\,. \tag{S2}$$

The density of condensate

$$n_{BEC}(\mathbf{k}) = (1-\delta_B)\,|\phi(\mathbf{k},t)|^2, \tag{S3}$$

is augmented by the $1-\delta_B$ factor when calculating observables to conserve overall particle number. The k-space fields here are normalised as

$$\begin{bmatrix} \widehat{\Psi}_B(\mathbf{k}) \\ \psi_B(\mathbf{k}) \\ \widetilde{\psi}_B(\mathbf{k}) \end{bmatrix} = \frac{1}{(2\pi)^3}\int d^3\mathbf{x}\, e^{-i\mathbf{kx}} \begin{bmatrix} \widehat{\Psi}_B(\mathbf{x}) \\ \psi_B(\mathbf{x}) \\ \widetilde{\psi}_B(\mathbf{x}) \end{bmatrix} \tag{S4}$$

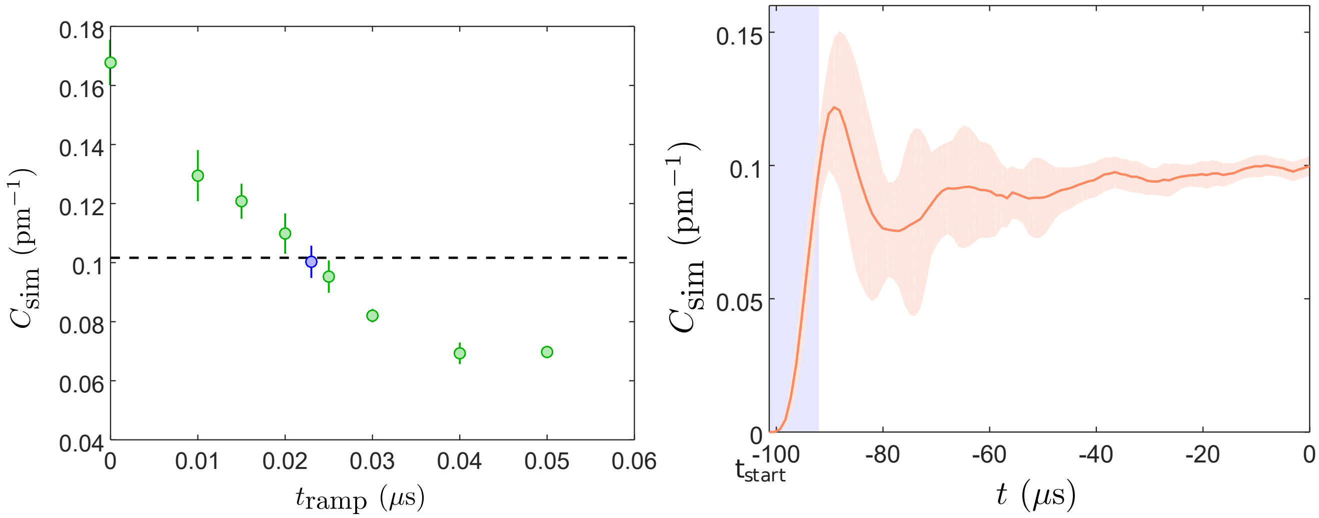

Figure S3. Details of initial state preparation, shown on the $N = 455852$ case. Upper panel: Calibration data. Shown is the calculated contact C_{sim} at the end of the initial state generation ($t = 0$), as a function of the quench ramp time t_{ramp}. The horizontal dashed line shows the LDA prediction $C = 0.102$ pm^{-1}, the blue data point the ensemble that was deemed to agree, and was used for subsequent simulations for $t > 0$. Lower panel: Evolution and stabilisation of the contact. The blue shaded area shows the duration of the ramp from t_{start} to $t_{\text{start}} + t_{\text{ramp}}$. Orange shading denotes the error bars. $\mathscr{S} = 4000$ trajectories in all cases.

Initial ensemble

A procedure for generating the equilibrium state has been developed in the Wigner representation[32,86–89]. Unfortunately we cannot use this directly, nor a direct Wigner representation of the quasiparticles because the condition required for correctness of the Wigner representation – that there are more particles than modes – is very far from being met[33,90,91] (In fact here we have about $\mathscr{O}(1000)$ particles in the depletion, and $\mathscr{O}(10^6)$ modes.). It is also unclear how to translate a local density formulation of depletion in a uniform section of gas to a positive-P ensemble without introducing discontinuities. Instead we turned to dynamically generating a state with the required quantum depletion.

We begin with a fully condensed ground state with $\psi_B = \widetilde{\psi}_B = 0$ and $\phi(\mathbf{x}) = \phi_0(\mathbf{x})$, the ground state of the Gross-Pitaevskii equation (21). The latter is obtained by imaginary time propagation of the GPE augmented with an appropriately chosen chemical potential μ according to (8) which sets the central density.

Our first attempt to generate the equilibrated quantum depleted state thus started with $\phi_0(\mathbf{x})$ and then adiabatically ramped the interaction from $g = 0$ to the experimental value while evolving the equations (21)–(23). This did not work for two reasons. Firstly, a very strong collective oscillation was induced, since the width of the Thomas-Fermi ground state depends strongly on g. Secondly, very long time evolutions succumb to excessive noise in the positive-P simulation and produce a state that is too noisy to be useful. Note that in the positive-P representation different ensembles can represent the same state but exhibit very different noisiness and practical usefulness[92,93].

The second attempt began with the Gross-Pitaevskii ground state and the target physical interaction strength g in the GPE (21), while slowly ramping the interaction strength in the Bogoliubov equations (22)–(23). This eliminates the main oscillations in the mean-field evolution. The least unwanted nonadiabatic disturbance occurs when g is ramped only within the projected part of the Bogoliubov evolution as per

$$i\hbar\frac{d\phi}{dt} = \mathscr{H}(g,\phi)\phi = (21) \tag{S5a}$$

$$i\hbar\frac{d\psi_B}{dt} = \mathscr{H}(g,\phi)\psi_B + \mathscr{P}_\perp\left\{g_B|\phi|^2\psi_B + g_B\phi^2\widetilde{\psi}_B^* + \sqrt{-ig_B}\,\phi\,\xi(\mathbf{x},t)\right\} \tag{S5b}$$

$$i\hbar\frac{d\widetilde{\psi}_B}{dt} = \mathscr{H}(g,\phi)\widetilde{\psi}_B + \mathscr{P}_\perp\left\{g_B|\phi|^2\widetilde{\psi}_B + g_B\phi^2\psi_B^* + \sqrt{-ig_B}\,\phi\,\widetilde{\xi}(\mathbf{x},t)\right\}. \tag{S5c}$$

with $g_B(t)$. However, over adiabatic timescales, this still introduced far too much noise in the Bogoliubov fields ψ_B, $\widetilde{\psi}_B$ to be useful.

To work around the problem, we take advantage of the fact that an instantaneous quantum quench of a weakly interacting condensate produces depletion with a similar time-integrated momentum profile $\int dt\, n_B(\mathbf{k},t) \propto 1/k^4$ to the ground state value, but with a somewhat higher depletion, as described in detail in[61,80]. Simulations show that non-instantaneous but rapid quenches produce lower amounts of quantum depletion, as shown in Figure S3 (left panel). To generate these ensembles, we used the following ramp:

$$g_B(t) = \begin{cases} \left(\frac{t-t_{\text{start}}}{t_{\text{ramp}}}\right) g & \text{when} \quad t < t_{\text{start}} + t_{\text{ramp}} \\ g & \text{when} \quad t \geq t_{\text{start}} + t_{\text{ramp}} \end{cases} \tag{S6}$$

starting at negative t_{start}, and evolving till $t = 0$. Thus t_{ramp} was the ramp time, and the remaining time: $|t_{\text{start}}| - t_{\text{ramp}}$ an equilibration time. We carried out a calibration like that shown in Figure S3 (left panel) for each set of cloud and trap parameters needed. Then for the initial *in situ* ensemble in the trap we chose the ensemble generated with the t_{ramp} that produces total quantum depletion in agreement with the *in situ* value (9) for the condensate ground state (like the blue data point in Figure S3, left panel). The value of t_{start} was -101μs for the weakly trapped cases ($\omega = 45 \times 425 \times 425$ Hz and $\bar{\omega} = 201$ Hz) and -198μs for the strongly trapped cases ($\omega = 71 \times 902 \times 895$ Hz and $\bar{\omega} = 393$ Hz) detailed in Table. S3. Figure S3 (right panel) shows an example of the evolution of the contact *in situ* during this initial state generation. Properties of the initial states are labelled (CT) and (ST) in Fig. 3 (a) (main text).

Determination of the contact and tail strengths

Much as in the experiment, the k-space density $n(\mathbf{k})$ in the simulations is very noisy in the asymptotic region of large $|\mathbf{k}|$, and a lot of averaging is needed to extract the contact. In light of the discussion on power laws, the exponent α was not fitted here, but we did calculate both the fit coefficients C_{sim} on the function $n_B(k) = C_{\text{sim}}/k^4$ with $\alpha = 4$ assumed and the count of particles in the tails, $N_{k_{\text{min}},k_{\text{max}}}$ as in Table S2, in order to have a systematic view of how correlated the two kinds of results are.

We proceed as follows: The simulations provide a density of depleted particles $n_B(\mathbf{k})$. Like with the experimental counts, we keep only density that is far enough away (ϕ_c, usually 60°) from the long axis of the cloud. The simulated system is axially symmetric around the long axis. We do not restrict counting to the vicinity of the vertical axis because here there are no detector irregularities to avoid, and this allows us to improve the signal to noise ratio.

The particle counts $N_B(\mathbf{k}) = n_B(\mathbf{k})/V$ are finely binned according to the absolute value of the momentum $k = |\mathbf{k}|$, giving total bin count N_k. The field is simulated on a square lattice in k space, in which each site corresponds to a k-space volume of $\Delta V_K = (2\pi)^3/V$ with real space volume V. Therefore the total bin volume V_k is obtained by binning the site volumes, and the mean density in each bin is N_k/V_k so $n_k = (2\pi)^3 N_k/V_k$. Each bin gives an estimate of the corresponding apparent contact:

$$c(k) = n_k k^4 = \frac{N_k}{V_k} k^4 (2\pi)^3. \tag{S7}$$

The statistical error estimate on $c(k)$, $\Delta c(k)$, is obtained by averaging subensembles, then using the central limit theorem (CLT) on the subensemble averages. Figure S4 shows example values and error estimates, while also demonstrating the difficulties involved.

We can see that for a significant range of k values the $c(k)$ estimate gives fluctuations around a constant value. However, several difficulties in extracting the overall trend are also evident. Low k values are not representative because the particles in the shaded region never emerge from the condensate and are not measured in the experiment. Therefore we remove data below a value k_{inner} from consideration. k_{inner} is chosen such that it does not include any depletion that would significantly overlap with the BEC in the final expanded cloud and be obscured by it.

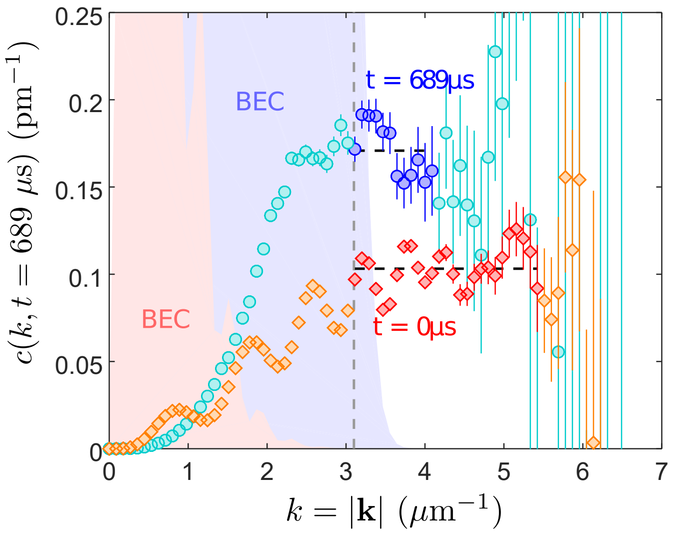

Figure S4. The extraction of contact from simulation data – shown by example of the $N = 455852$ trap release (CE) simulation. Blue/cyan data: after expansion at $t = 689\mu$s, Red/orange: in situ initial condition at $t = 0$. Each data point corresponds to estimation of $c(k)$ given by (S7) using the density of particles in the Bogoliubov field $\widehat{\Psi}_B(\mathbf{k})$ in a radial bin centred at $k = |\mathbf{k}|$. Error bars give the statistical error from an ensemble of $\mathscr{S} = 4000$ trajectories. Blue and red points are the fitted part of the data. The vertical dashed line indicates k_{inner}, while the shaded region on the left shows the corresponding scaled density $c_{\text{BEC}}(k) = n_{\text{BEC}}(k)k^4$ that would be calculated from the condensate field $\phi(\mathbf{k})$. The horizontal dashed lines indicate the final estimates of C_{sim} extracted from the data.

This corresponds also to the energy at which the quasiparticle spectrum becomes particle-like, since the mean field energy in the centre of the trapped cloud is responsible for both effects.

High k values, on the other hand, suffer from statistical error sufficiently high as to make them useless, and so we also choose a maximum k_{outer} value, and only use $k \in [k_{\text{inner}}, k_{\text{outer}}]$ to extract the contact estimate. The extent of the useless high k region changes with time during the simulation. To systematically adapt our fitting region to this, we choose k_{outer} according to the calculated statistical error such that

$$\Delta c(k,t) \leq \Delta_{\max} \quad \forall k \leq k_{\text{outer}}(t), \tag{S8}$$

and $\Delta_{\max}$ is chosen once for all times in a simulation. This gives a time-dependent fitting region $k \in [k_{\text{inner}}, k_{\text{outer}}(t)]$. The final contact estimate $C_{\text{sim}}(t)$ is the mean of all $c(k,t)$ values in the fitting range. Error bars on $C_{\text{sim}}(t)$, $\Delta C_{\text{sim}}(t)$, are obtained from a CLT estimate of the error in the mean of the points used, after binning enough neighbouring points to encompass the autocorrelation (i.e. so that neighbouring bins are uncorrelated). The $\Delta_{\max}$ is chosen low enough so that the statistical error in individual points $\Delta c(k,t)$ at the large k end does not put off the overall contact estimation $C_{\text{sim}}(t) \pm \Delta C_{\text{sim}}(t)$. Operationally we choose the value of $\Delta_{\max}$ at which the error introduced by adding successive high k points introduces more uncertainty than the reduction thanks to a larger ensemble.

We also found that the relative factors between Eqn. (14) and the simulated tails (i.e. $C_{\text{sim}}/\mathscr{C}$ and $\mathscr{A}_{\text{sim}}$) depend on choice of cutoff angle ϕ_c. We find that apparent tail strengths C_{sim} and $\mathscr{A}_{\text{sim}}$ are larger for smaller collection regions that are more tightly concentrated about the strong trapping axis, whereas larger collection angles (that include areas closer to the weak axis) produce lower apparent tail strengths. Data on $\mathscr{A}_{\text{sim}}$ are shown in Table S2 while C_{sim} was 1.6(1), 1.9(2), and 2.2(3) times $\mathscr{C}$ for ϕ_c values of 60, 70, and 80 degrees, respectively.

n_oN ($10^6\mu$m^{-3})	$k_{\min}$ $k_{\max}$ (μm^{-1})		ϕ_c (deg)	$N_{k_{\min},k_{\max}}$ final	ratio $\mathscr{A}_{\text{sim}}$ final/in situ
Rapid release from trap (CE)					
5.237(16)	2.0	3.5	60	193(8)	1.38(5)
12.09(6)	2.25	3.5	60	362(10)	1.43(4)
6.86(3)	2.75	4.0	60	163(4)	1.45(4)
19.90(8)	3.0	4.0	40	423(8)	1.37(3)
19.90(8)	3.0	4.0	50	408(8)	1.56(3)
19.90(8)	3.0	4.0	60	368(7)	1.80(4)
19.90(8)	3.0	4.0	70	282(6)	2.00(4)
19.90(8)	3.0	4.0	80	157(5)	2.19(6)
Rapid release, spherical (SE)					
5.35(2)	2.0	3.25	60	120(9)	0.92(6)
12.23(6)	2.0	3.0	60	256(11)	1.05(4)
20.38(10)	3.0	4.0	60	232(10)	1.10(5)
Slow ramp down of trap (CS)					
5.237(16)	2.0	3.5	60	55(10)	0.37(6)
12.09(6)	2.0	3.5	60	103(21)	0.31(5)
6.86(3)	2.5	4.0	60	50(8)	0.34(6)
19.90(0)	2.5	4.0	60	181(14)	0.54(4)

Table S2. Tail strength data in simulations. Based on final times in the simulations described in Table S3, referenced by the value of n_0N. $N_{k_{\min},k_{\max}}$ is calculated as per Eqn (14) in the main text. The ratio $\mathscr{A}_{\text{sim}}$ of tail strength in the expanded cloud compared to Tan theory predictions is obtained by dividing $N_{k_{\min},k_{\max}}$ at the final time by its value in situ at $t=0$. $k_{\max}$ is chosen to still contain all 4π steradians inside the square lattice, while $k_{\min}$ to avoid overlap with the expanding condensate.

Trap at $t=0$ ω (Hz)	Peak density n_0 at $t=0$ (μm^{-3})	N	n_oN ($10^6\mu$m^{-3})	timescale (μs)	Time t (μs)	$C_{\text{sim}}(t)$ (pm^{-1})	$C_{\text{sim}}(t)/C_{\text{sim}}(0)$	$N_B(t)$	k_{inner}–k_{outer} (μm^{-1})	$\Delta_{\max}$ (μm^{-1})
In situ initial state (CT,ST):										
$425\times425\times45$	16.82(5)	311360	5.237(16)	t_B=19.8	0	0.0260(13)	–	1391(7)	2.1–4.2	0.025
$425\times425\times45$	21.32(10)	567180	12.09(6)	t_B=15.8	0	0.060(5)	–	2860(13)	2.2–4.75	0.032
$902\times895\times71$	32.17(13)	213293	6.86(3)	t_B=8.5	0	0.034(4)	–	1324(6)	2.9–4.9	0.020
$902\times895\times71$	43.66(18)	455852	19.90(8)	t_B=9.3	0	0.100(3)	–	3047(12)	3.25–5.4	0.032
$201\times201\times201$	16.88(6)	316766	5.35(2)	t_B=19.8	0	0.025(3)	–	1396(7)	2.1–4.1	0.025
$201\times201\times201$	21.32(10)	573650	12.23(6)	t_B=14.2	0	0.060(9)	–	2741(11)	2.2–4.15	0.04
$393\times393\times393$	44.15(21)	461514	20.38(10)	t_B=7.7	0	0.101(5)	–	3107(12)	3.0–5.0	0.025
Rapid release from trap (CE,SE), $\tau_{\text{release}}=37.5\mu$s:										
$425\times425\times45$	16.82(5)	311360	5.237(16)	τ_{release}	1583	0.0386(22)	1.49(11)	910(20)	2.1–2.8	0.020
$425\times425\times45$	21.32(10)	567180	12.09(6)	τ_{release}	1346	0.090(7)	1.51(17)	1880(40)	2.3–3.05	0.020
$902\times895\times71$	32.17(13)	213293	6.86(3)	τ_{release}	689	0.057(5)	1.69(14)	950(20)	2.8–3.7	0.03
$902\times895\times71$	43.66(18)	455852	19.90(8)	τ_{release}	689	0.171(10)	1.66(12)	2300(40)	3.1–4.1	0.06
$201\times201\times201$	16.82(5)	316766	5.35(2)	τ_{release}	1900	0.025(3)	1.02(15)	780(30)	1.9–2.65	0.025
$201\times201\times201$	21.32(10)	573650	12.23(6)	τ_{release}	1900	0.066(7)	1.11(20)	1630(50)	2.3–3.15	0.05
$393\times393\times393$	44.15(21)	461514	20.38(10)	τ_{release}	973	0.110(5)	1.09(8)	1780(50)	3.1–3.8	0.05
Slow ramp down of trap (CS):										
$425\times425\times45$	16.82(5)	311360	5.237(16)	t_{ramp}=2375	2375	0.010(3)	0.40(12)	1750(140)	1.9–3.45	0.030
$425\times425\times45$	21.32(10)	567180	12.09(6)	t_{ramp}=2375	2375	0.020(3)	0.35(7)	2700(300)	2.0–3.9	0.040
$902\times895\times71$	32.17(13)	213293	6.86(3)	t_{ramp}=1216	1216	0.011(6)	0.3(2)	1520(150)	2.5–4.2	0.040
$902\times895\times71$	43.66(18)	455852	19.90(8)	t_{ramp}=1216	1216	0.044(5)	0.45(6)	3800(400)	2.5–4.1	0.035
Instantaneous switch-off of the trap:										
$902\times895\times71$	43.66(18)	455852	19.90(8)	t_{ramp}=0	689	0.0187(6)	1.80(6)	2380(40)	3.1–4.05	0.05

Table S3. Main simulation data and parameters for fitting of k^{-4} tail amplitude C_{sim}. The time t for which data are calculated is counted relative to the start of the trap release. Abbreviations (CT,CE,...) as in Figure 3a of the main text. The range k_{inner}–k_{outer} here was chosen as explained in the text and used to obtain the estimate and uncertainty for C_{sim}. In all cases, $\mathscr{S}=4000$ trajectories averaged. N_B is the number of Bogoliubov field particles as per (S1).

Toy two-mode model of escape

In a uniform gas in the Bogoliubov approximation, the $\widehat{a}_k$ mode is coupled only to $\widehat{a}^\dagger_{-k}$ and the condensate. The Bogoliubov-de Gennes equations for these modes can then be written[81]

$$\frac{d}{dt}\begin{bmatrix} \widehat{a}_k(t) \\ \widehat{a}^\dagger_{-k}(t) \end{bmatrix} = -\frac{i}{\hbar}\begin{bmatrix} \hbar^2k^2/2m + 2gn(t) - \mu(t) & gn(t) \\ -gn(t) & -\hbar^2k^2/2m - 2gn(t) + \mu(t) \end{bmatrix}\begin{bmatrix} \widehat{a}_k(t) \\ \widehat{a}^\dagger_{-k}(t) \end{bmatrix}. \tag{S9}$$

Notice the provision for a time-dependent background condensate density $n(t)$. We stay in the real-particle basis rather than the Bogoliubov quasiparticles $\widehat{b}_k$ which allows to avoid calculating time-dependent changes of the coefficients u_k, v_k. The chemical potential is $\mu(t) = gn(t)$[81].

The equations (S9) can be used as input for equations of motion of the low order moments $\rho(k) = \langle \widehat{a}^\dagger_{\pm k}\widehat{a}_{\pm k}\rangle$, $A(k) = \langle \widehat{a}_k\widehat{a}_{-k}\rangle$, and $A^*(k) = \langle \widehat{a}^\dagger_k\widehat{a}^\dagger_{-k}\rangle$, for example $d\rho(k)/dt = \langle (d\widehat{a}^\dagger_k/dt)\widehat{a}_k\rangle + \langle \widehat{a}^\dagger_k(d\widehat{a}_k/dt)\rangle$. Assuming equal initial occupations $\rho(k,0) = \rho(-k,0)$, one obtains an evolution equation for two coupled quantities $\rho(k,t)$ and $A(k,t) = A_r(k) + iA_i(k)$. It is

$$\frac{d}{dt}\begin{bmatrix} \rho(k,t) \\ A_r(k,t) \\ A_i(k,t) \end{bmatrix} = \frac{1}{\hbar}\begin{bmatrix} 0 & 0 & -2gn(t) \\ 0 & 0 & 2(\hbar^2k^2/2m - gn(t)) \\ -2gn(t) & -2(\hbar^2k^2/2m - gn(t)) & 0 \end{bmatrix}\begin{bmatrix} \rho(k,t) \\ A_r(k,t) \\ A_i(k,t) \end{bmatrix} + \begin{bmatrix} 0 \\ 0 \\ -gn(t) \end{bmatrix}. \tag{S10}$$

The initial conditions are $\rho(k,0) = v_k^2$, $A(k,0) = v_k u_k$, corresponding to the Bogoliubov ground state. Taking $n(t)$ constant one obtains the solution (16).

A more realistic model is obtained by estimating the $n(t)$ that particles in the k mode experience as the trap is released and the condensate expands. Hydrodynamic expansion of a condensate with a Thomas-Fermi profile suddenly released from the trap[94] gives a self-similar expansion with widths approximated by $W_j(t) = W_j(0)\sqrt{1+(\omega_j t)^2}$. The initial widths being the Thomas Fermi radii in the trap $R_j = (1/\omega_j)\sqrt{2gn_0)/m}$, $j = \{x,y,z\}$. From this, and assuming $\omega_y = \omega_z = \omega_\perp = \bar{\omega}\lambda^{1/3}$ (approximately true in our setup) the condensate density at any point in the cloud decays as

$$n_c(r,t) = \frac{n_c(r,0)}{(1+\omega_\perp^2 t^2)\sqrt{1+\omega_x^2 t^2}}, \qquad \text{where} \qquad n_c(r,0) = \begin{cases} n_0\left[1-(r/R_\perp)^2\right] & \text{if } r < R_\perp \\ 0 & \text{if } r \geq R_\perp \end{cases} \tag{S11}$$

Escape is much easier, however, for atoms near the edge of the cloud than in the centre, because they can avoid reabsorption by faster free-flight from the high density region. We categorise the initial position of the studied section of the gas with the quantity

$$R_0 = \frac{\sqrt{y^2+z^2}}{R_\perp} = \frac{\omega_\perp\sqrt{y^2+z^2}}{\sqrt{2gn_0/m}}. \tag{S12}$$

The initial density is then

$$n(0) = \begin{cases} n_0(1-R_0^2) & \text{if } R_0 \leq 1 \\ 0 & \text{if } R_0 > 1 \end{cases} \tag{S13}$$

The motion of the atoms is estimated by assuming they travel in the outward direction at velocity $\hbar k/m$ relative to the hydrodynamic expansion (which also accelerates them to some degree). Hence their position is estimated as

$$r(t) = R_0R_\perp\sqrt{1+\omega_\perp^2 t^2} + \frac{\hbar k t}{m}. \tag{S14}$$

The condensate at this position comes from hydrodynamic expansion of the initial condensate at position

$$r_{c0}(t) = \frac{r(t)}{\sqrt{1+\omega_\perp^2 t^2}} = R_0R_\perp + \frac{\hbar k t}{m\sqrt{1+\omega_\perp^2 t^2}}. \tag{S15}$$

The local density at position r at time t is then $n_c(r_{c0}(t),t)$ according to (S11).

Finally, to roughly include ramp time in the analysis, consider the density in a trapped gas that adiabatically follows the trap evolution $\omega_j(t) = \omega_j(0)e^{-t/\tau_{\text{release}}}$. The overall density for a piece of gas that stays in the same relative position in the cloud will then follow

$$n_c^{\text{adiabatic}}(t) = n(0)e^{-3t/\tau_{\text{release}}}. \tag{S16}$$

To incorporate this effect into the model it is combined (somewhat ad hoc) with the expression $n_c(r_{c0}(t),t)$ for instantaneous release to give the final estimate of local density for the k momentum escaping atoms used in (S10) as

$$n(t) = n(0)e^{-3t/\tau_{\text{release}}} + (1 - e^{-3t/\tau_{\text{release}}})\, n_c(r_{c0}(t),t). \tag{S17}$$

with the help of (S11) and (S15).
